# Simplified *in vitro* generation of human gastruloids for modelling early development

**DOI:** 10.1242/dev.205719

**Published:** 2026-10-23

**Authors:** Takuya Azami, E. Elizabeth Patton, Jennifer Nichols

**Affiliations:** ^1^ MRC Human Genetics Unit, Institute of Genetics and Cancer, University of Edinburgh, Edinburgh EH4 2XU, UK; ^2^ Cancer Research UK Scotland Centre, Institute of Genetics and Cancer, University of Edinburgh, Edinburgh EH4 2XU, UK

**Keywords:** Human pluripotent stem cells, Gastruloids, Lineage segregation, Axis elongation, Optimization

## Abstract

The purpose of this study was to optimize the efficiency, cost and reproducibility of human gastruloid formation by testing and adjusting individual parameters using four human pluripotent stem cell lines available from the UK Stem Cell Bank. First, commercially sourced culture medium was replaced with a home-made defined recipe, known as ‘N2B27’, into which specific reagents can be titrated. By reducing the concentration of activin A to 15% of the original protocol, efficient elongation of aggregated embryoid bodies was achieved. Also, titrating initial cell density and delaying brief exposure to GSK3 inhibitor until the onset of cell aggregation in individual wells was advantageous. Efficiency of formation of early gastruloids exhibiting the expected regionalization of the three embryonic germ layers was further enhanced by addition of TGFβ inhibitor. The optimization steps presented thus provide a simplified, robust and relatively economical protocol for consistent generation of elongated gastruloids from human pluripotent stem cells. This streamlined method improves accessibility and reproducibility, also providing an adaptable platform to investigate fundamental principles of early human development.

## Teaser

**Summary:** We present an optimized protocol for human gastruloid production that should enhance efficiency, reproducibility and affordability for future *in vitro* studies into early human post-implantation development.

## INTRODUCTION

Human gastrulation begins with the differentiation of primitive streak (PS) cells around embryonic day (E)14-E15, Carnegie stages (CS)6-CS7, and concludes by ∼E21 (CS9), culminating in the formation of the three germ layers: ectoderm, mesoderm and endoderm (Hertig et al., 1956). This developmental phase is also characterized by the spatial organization of distinct cell types along the embryonic body axes. Nevertheless, our understanding of gastrulation in human remains relatively limited owing to the practical and ethical inaccessibility of early post-implantation human embryos (Rossant, 2024). Gastruloids, three-dimensional models for early mammalian post-implantation development and gastrulation, first developed using mouse embryonic stem cells (mESCs) (van den Brink et al., 2014), enable study of axial elongation and germ layer differentiation. Gastruloids made from human pluripotent stem cells (hPSCs) provide an exciting system with which to investigate human development post-implantation, but required modification to the original mouse protocols and optimization for individual hPSC lines (Moris et al., 2020; Olmsted and Paluh, 2021).

In the case of mouse gastruloids, naïve pluripotent mESCs are aggregated and differentiated to a post-implantation epiblast-like state for the first 48 h of the protocol (Beccari et al., 2018; Turner et al., 2017). Gastrulation is then stimulated using the GSK3β inhibitor, CHIR99021 (CHIR), which in this context acts as a WNT agonist. In contrast, conventional hPSCs are generally maintained in a ‘primed’ pluripotent state, representative of the post-implantation epiblast, rendering them rapidly responsive to gastrulation-inducing signals. The original protocols pioneering formation of human gastruloids depended upon pre-CHIR treatment (Hamazaki et al., 2024; Moris et al., 2020; Neupane et al., 2025; Olmsted and Paluh, 2021); however, how CHIR pretreatment impacts gastruloid generation and reproducibility has not been determined. In this study, we set out to optimize and simplify the conditions for human gastruloid formation. We modified the protocol to include ‘in house’ formulated medium in which we tested multiple variables using distinct hPSC lines with the aim of improving germ layer specification and consistency. We demonstrate that cell density during CHIR stimulation crucially influences gastruloid induction, particularly the balance between FOXA2^+^ endoderm and TBXT^+^ mesoderm populations. Low-density cultures favour FOXA2^+^ endoderm differentiation, whereas high-density conditions suppress endodermal specification. Based on these findings, we have established a human gastruloid protocol independent of pre-CHIR treatment using defined culture conditions. This improved method enables highly reproducible generation of robust elongated gastruloids, with the added advantage of reducing cost by replacing commercial medium with N2B27 formulated in-house as well as significantly lower concentrations of activin A.

## RESULTS AND DISCUSSION

### Low activin A enhances human gastruloid generation and consistency

We first attempted to generate human gastruloids using a defined culture medium: N2B27, instead of the commercially produced Nutristem or Essential 8, supplemented with activin A (20 ng/ml), FGF2 (12.5 ng/ml) and XAV939 (WNT inhibitor, 2 μM) (hereafter referred to as ‘A20FX’) (Kinoshita et al., 2021; Rostovskaya et al., 2019). This formulation enables modulation of signalling pathways by adding or withdrawing individual components. We used four hPSC lines, derived between 1998 and 2016 using distinct protocols. H9 was one of the original lines produced by outgrowth of whole blastocysts in medium containing FGF4 and serum (Thomson et al., 1998), which produces flat, epithelialized colonies representative of the primed state of pluripotency (Nichols and Smith, 2009). Shef6 was derived directly from blastocysts using a microdrop culture system (Aflatoonian et al., 2010). The HNES1 and HNES3 lines were generated from inner cell masses (ICM) isolated by immunosurgery (Solter and Knowles, 1975) from D6 human blastocysts using anti-human antiserum, disaggregated and physically separated in culture medium optimised to maintain self-renewal in the naïve state of pluripotency (Guo et al., 2016; Takashima et al., 2014). For the current study, HNES1 and HNES3 cells were converted to the primed state via capacitation, a phenomenon readily achieved by addition of the tankyrase inhibitor XAV939, or the porcupine inhibitors IWP2 or C59 to the culture medium (Rostovskaya et al., 2019) (see Materials and Methods). Primed (p)H9, Shef6, pHNES1 and pHNES3 were cultured in A20FX for at least three passages before generation of gastruloids.

As cell density during the pre-CHIR treatment before aggregation has been shown to be crucial for human gastruloid production (Moris et al., 2020), we plated cells at various densities in 12-well plates according to our initial culture regime (Fig. 1A). Under these conditions, A20FX-cultured hPSCs produced disorganised and degenerating gastruloids at all tested plating densities (Fig. 1B,C). Gastruloid morphology was classified based on axial length and elongation index (EI), defined as the ratio of the total axial length to the diameter of the largest inscribed circle within the gastruloid (Guiet et al., 2020) (see Materials and Methods; Fig. 1C; Fig. S1A,B). We titrated the activin A concentration to 3 ng/ml (A3FX), since capacitated hPSCs can be maintained efficiently in low activin A-containing medium (Rostovskaya et al., 2019) and low activin A concentration enables maintenance of pluripotency of hPSCs or induced (i)PSCs (Tomizawa et al., 2011; Xiao et al., 2006). Elongated gastruloids were formed from both pHNES1 and pH9 cells in A3FX culture; however, the efficiency was dependent upon plating density, with the highest success observed at 6×10^4^ cells per 12-well plate (Fig. 1B,C; Fig. S1B). Gastruloids from both cell lines exhibited markers of mesoderm (TBXT), endoderm (GATA6) and epiblast (SOX2) (Fig. 1D). These experiments confirmed the importance of optimising cell density and concentrations of growth factors for consistent formation of elongated human gastruloids.

**Fig. 1.**
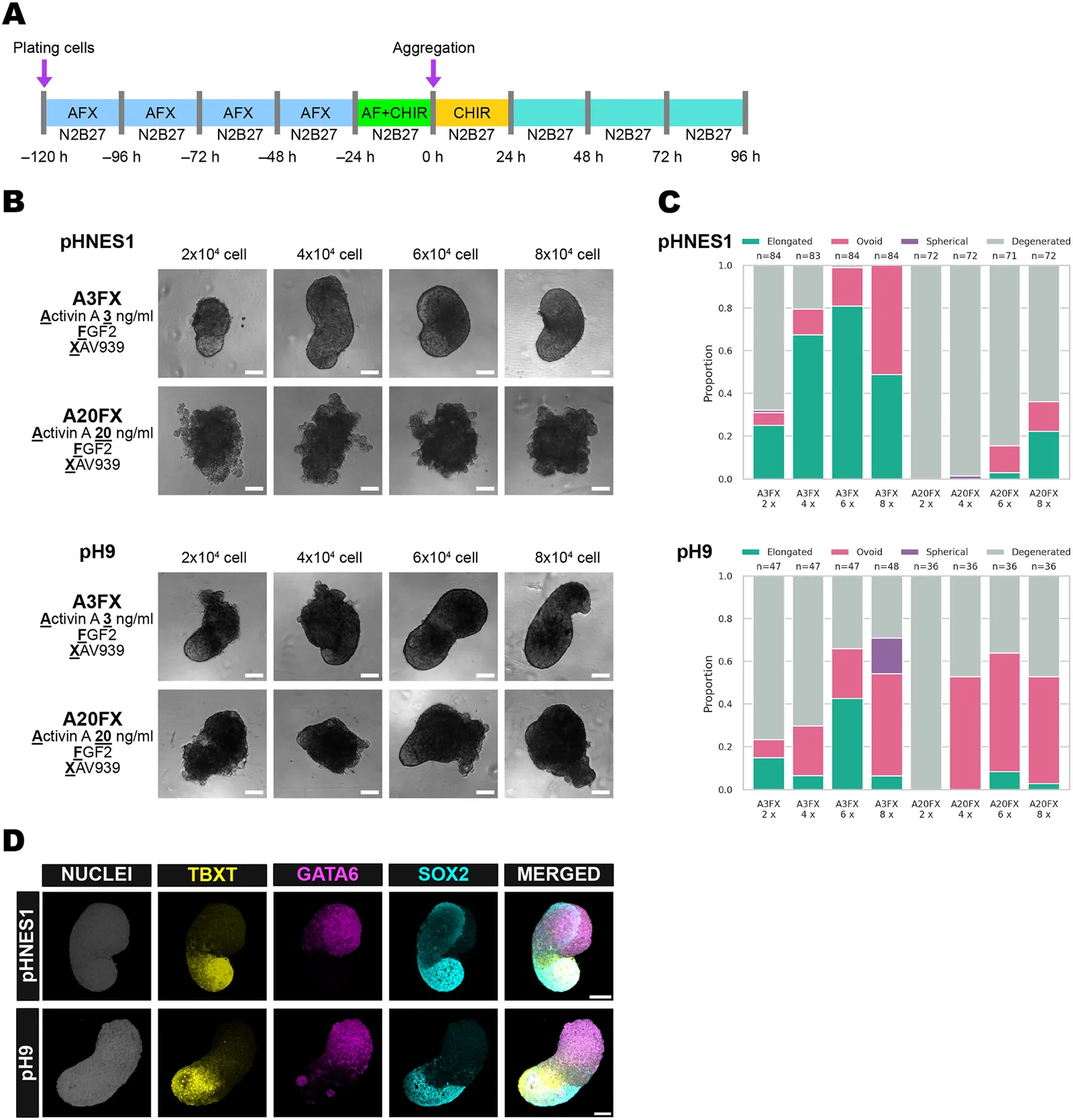
**Activin A concentration and cell culture density determine the efficiency of elongated gastruloid formation.** (A) Schematic of protocol for making human gastruloids, adapted from Moris et al. (2020). (B) Representative brightfield images for gastruloids generated from A3FX or A20FX cultured pHNES1 and pH9 cells plated at 2×, 4×, 6× and 8×10^4^ cells/12-well plate. (C) Proportion of elongated, ovoid, spherical and degenerated morphology from pHNES1 and pH9 hPSCs. Plots represent pooled data from independent experiments (pHNES1 A3FX, *n*=4; pHNES1 A20FX, *n*=3; pH9 A3FX, *n*=3; pH9 A20FX, *n*=2). The number of gastruloids analysed (*n*) for each condition is indicated above the corresponding plot. Results from individual experiments are shown in Fig. S1B. (D) Immunofluorescence for TBXT, GATA6, and SOX2 in gastruloids generated by indicated conditions. Scale bars: 100 μm.

### The balance between proportions of TBXT^+^, SOX2^+^ and FOXA2^+^ cells is critical for human gastruloids

As cell density before CHIR treatment appeared important to form elongated human gastruloids, we immunostained cells cultured in monolayers at various densities after CHIR treatment to estimate the effect on subsequent lineage proportions (Fig. 2A,B). Expression of the endoderm marker FOXA2 (Burtscher and Lickert, 2009; Goldman et al., 2014) was preferentially induced in low density culture (2 or 4×10^4^ cells) (Fig. 2C-E; Fig. S2). TBXT- and SOX2-expressing cells were distributed randomly at low density. In contrast, at high-density, FOXA2 expression was suppressed and TBXT^+^ cells were located at the edge of colonies, while SOX2^+^ cells localised more centrally following 96 h of pre-culture before CHIR treatment (Fig. 2C-F; Fig. S2). This expression pattern is similar to 2D micropattern gastruloids, localising SOX2 at the centre and TBXT at the periphery (Etoc et al., 2016; Martyn et al., 2019a,b; Yoney et al., 2018). Although both micropatterns and pre-CHIR treatment induce gastrulation, the micropattern protocol includes BMP4 to activate WNT signalling. In high-density hPSC colonies, cellular responses to TGFβ ligands were spatially restricted to the colony edge, since dense epithelialisation leads to the re-localisation of TGFβ receptors to the basolateral surface, where they are shielded from apical ligands by tight junctions (Etoc et al., 2016). In the present study, both FOXA2 and TBXT expression were suppressed by high density culture in A3FX (Fig. 2C-F; Fig. S2B).

**Fig. 2.**
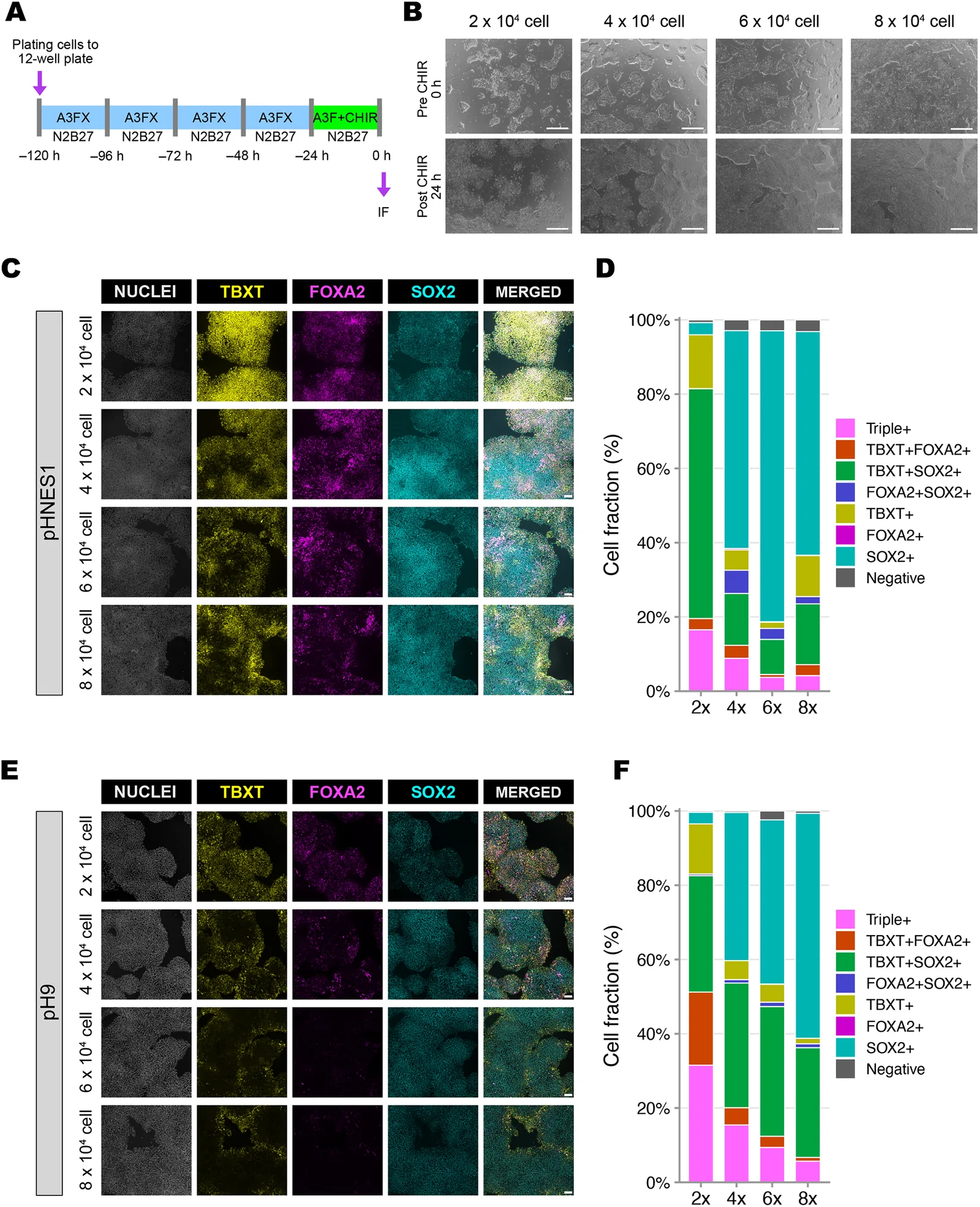
**Culture density influence on the balance of TBXT, SOX2 and FOXA2 expression.** (A) Schematic for the culture and pre-CHIR treatment of hPSCs. (B) Representative brightfield images of pHNES1 PSCs before and after pre-CHIR from various starting densities. (C) Immunofluorescence for TBXT, FOXA2 and SOX2 in pre-CHIR-treated pHNES1 PSCs. (D) Stacked bar charts showing the proportion of cells positive for TBXT, FOXA2 and SOX2 in pre-CHIR-treated pHNES1 PSCs. (E) Immunofluorescence for TBXT, FOXA2 and SOX2 in pre-CHIR-treated pH9 PSCs. (F) Stacked bar charts showing the proportion of cells positive for TBXT, FOXA2 and SOX2 in pre-CHIR-treated pH9 PSCs. For D and F, cells were classified into eight mutually exclusive categories based on normalized fluorescence intensity. Data represent the percentage of total cells per condition. Scale bars: 100 μm.

FOXA2 was barely visible in A20FX cultures at high density, and TBXT was reduced as density increased (Fig. S2C). These data indicate that high cell density in culture conditions before CHIR treatment maintains cells in an epiblast-like state, with reduced differentiation towards endoderm and mesoderm before the aggregation stage. Since cell density increases over the culture period, we performed pre-CHIR treatment 1-day earlier (Fig. S3A), at day 3 of culture, to scrutinise gene expression in both A3FX (Fig. S3B) and A20FX (Fig. S3C). Notably, TBXT- and FOXA2-expressing cells were distributed across colonies at all cell densities in A3FX (Fig. S3B). On the other hand, FOXA2 was not strongly induced in A20FX culture at day 3 pre-CHIR treatment (Fig. S3C). Since cell density determines the fate choice between surface ectoderm and amnion from hPSC (Nakanoh, 2025; Nakanoh et al., 2024), our findings provide further insight to develop efficient differentiation strategies to expand specific lineages from hPSCs.

To determine whether WNT activation is perturbated by cell density, we stained cultures for the WNT target LEF1, and observed strong induction at low density in A3FX for 4 days (Fig. S4A-C) before CHIR, but expression was suppressed in high density and A20FX cultures (Fig. S4B,C). Taken together, these results demonstrate that the generation of elongated gastruloids via pre-CHIR treatment is dependent on the balance of activin concentration and optimal cell density of hPSC.

### Gastruloid generation without pre-CHIR treatment

So far, our data suggest that hPSC cultures post-CHIR treatment are heterogeneous for endoderm, mesoderm and neuronal cell states, which are dynamic and sensitive to cell density and other conditions. We considered that aggregation from these mixed cell populations may contribute to stochastic variability of gastruloids, and that CHIR itself may be contributing to this heterogeneity. To address this, we attempted to generate gastruloids without pre-CHIR treatment (Fig. 3A), inspired by other related organoid protocols (Meijer et al., 2025; Rito et al., 2025; Sanaki-Matsumiya et al., 2022). To promote single-cell survival of hPSC and aggregate formation, we included CEPT cocktail (Chen et al., 2021; Ryu et al., 2024; Tristan et al., 2023), consisting of Chroman I, Emricasan, Polyamine supplement and trans-ISRB, instead of ROCK inhibitor. In either A3FX or A20FX, there was no apparent difference in the morphology of pHNES1 cells (Fig. 3B), but a slight increase in FOXA2^+^ cells was observed in A3FX cultures (Fig. 3C). Using A3FX and direct aggregation, elongating gastruloids could be formed more efficiently than those cultured in A20FX from pHNES1 cells (Fig. 3D,E; Fig. S5A,B). These gastruloids expressed the expected lineage markers, with TBXT and SOX2 localized to the posterior region and GATA6 to the anterior (Fig. 3F), an expression pattern similar to that observed in pre-CHIR-treated gastruloids (Fig. 1D).

**Fig. 3.**
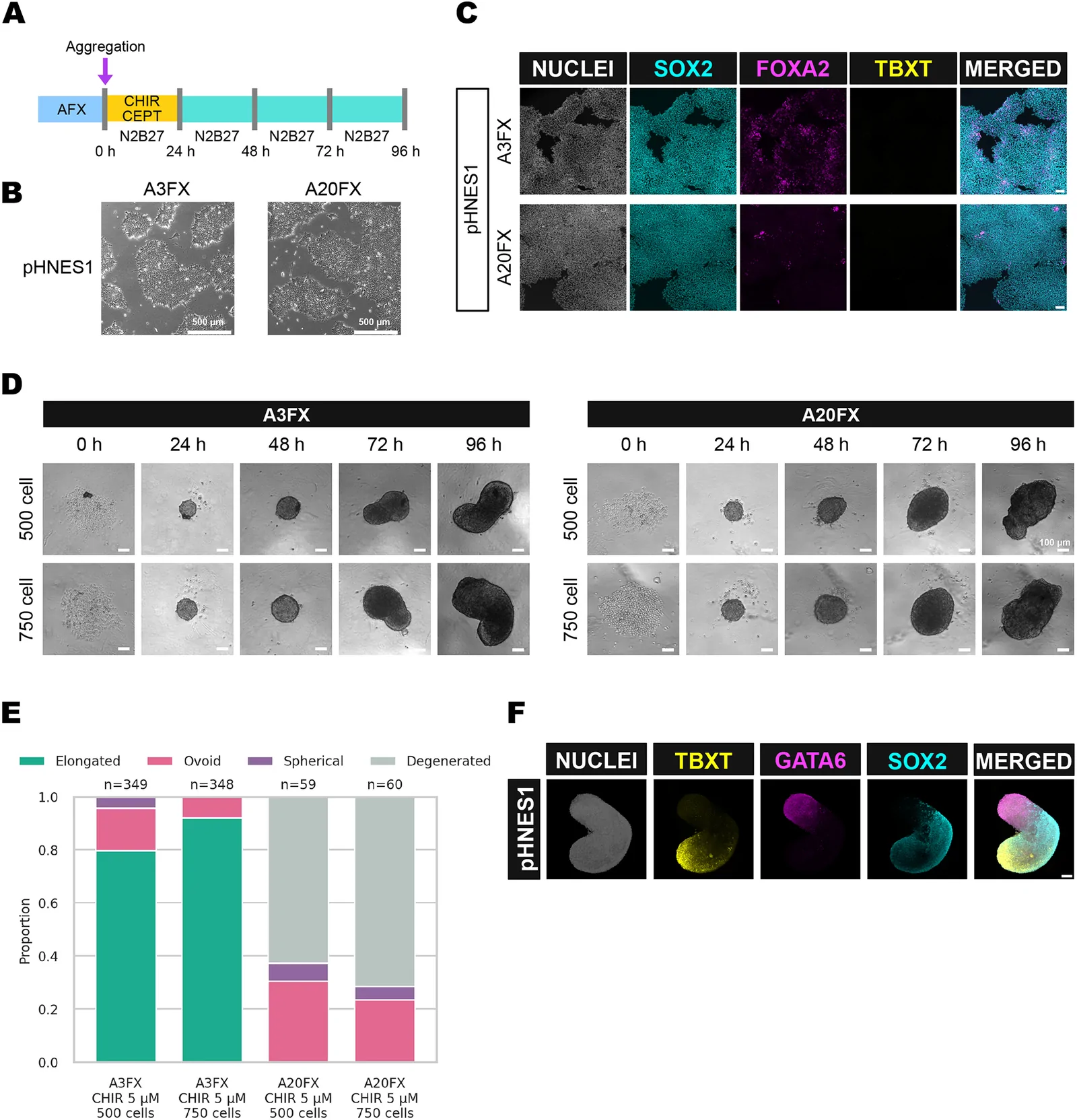
**Gastruloid differentiation via direct aggregation protocol.** (A) Schematic for the protocol for making gastruloids without pre-CHIR treatment, but including CHIR and CEPT cocktail after aggregation. (B) Representative brightfield images for A3FX- and A20FX-cultured pHNES1 hPSCs before making gastruloids. (C) Immunofluorescence for SOX2, FOXA2 and TBXT in A3FX- and A20FX-cultured pHNES1 hPSCs. (D) Representative images of gastruloid differentiation over the time course in A3FX or A20FX. (E) Proportion of elongated, ovoid, spherical and degenerated morphology from pHNES1 hPSCs. Plot represents a pool of 12 (A3FX, 500 cell), 11 (A3FX, 750 cells) and 3 (A20FX, 500 cells and 750 cells) independent experiments. The number of gastruloids (*n*) for each condition is indicated above the corresponding plot. (F) Immunofluorescence for TBXT, GATA6 and SOX2 of gastruloids generated from A3FX-cultured pHNES1 hPSCs. Scale bars: 500 μm (B); 100 μm (C,D,F).

Although exhibiting apparently similar appearance in monolayer culture compared with pHNES1 (Fig. 3B,C; Fig. S6A,B), pH9 cells were less efficient and more variable during gastruloid elongation when using the direct aggregation protocol (Fig. S6C-H, Fig. S7). To improve elongation efficiency, we introduced the TGFβ inhibitor SB431542 (SB43) after aggregation (Fig. 4; Fig. S6C), as SB43 has previously been used to promote gastruloid formation from H9 hPSCs (Hamazaki et al., 2024). SB43 significantly improved the efficiency of elongated gastruloid formation from H9 hPSCs cultured in A3FX; in A20FX, most aggregates became ovoid when SB43 was added (Fig. S6D,E, Fig. S7). Interestingly, although the consistency of shape and integrity of the H9 gastruloids was improved with SB43, GATA6 and SOX17 expression were absent (Fig. S6F,G). FOXA2 expression was induced following CHIR stimulation in 2D culture, more strongly in pHNES1 than pH9 cells (Fig. 2C-F), whereas GATA6 and SOX17 were reduced in SB43-treated pH9 compared with CHIR-treated gastruloids and monolayer culture (Fig. S6F-H), suggesting that endoderm differentiation is not fully specified at 24 h of differentiation and can be inhibited by SB43 treatment between 24 and 48 h after aggregation.

**Fig. 4.**
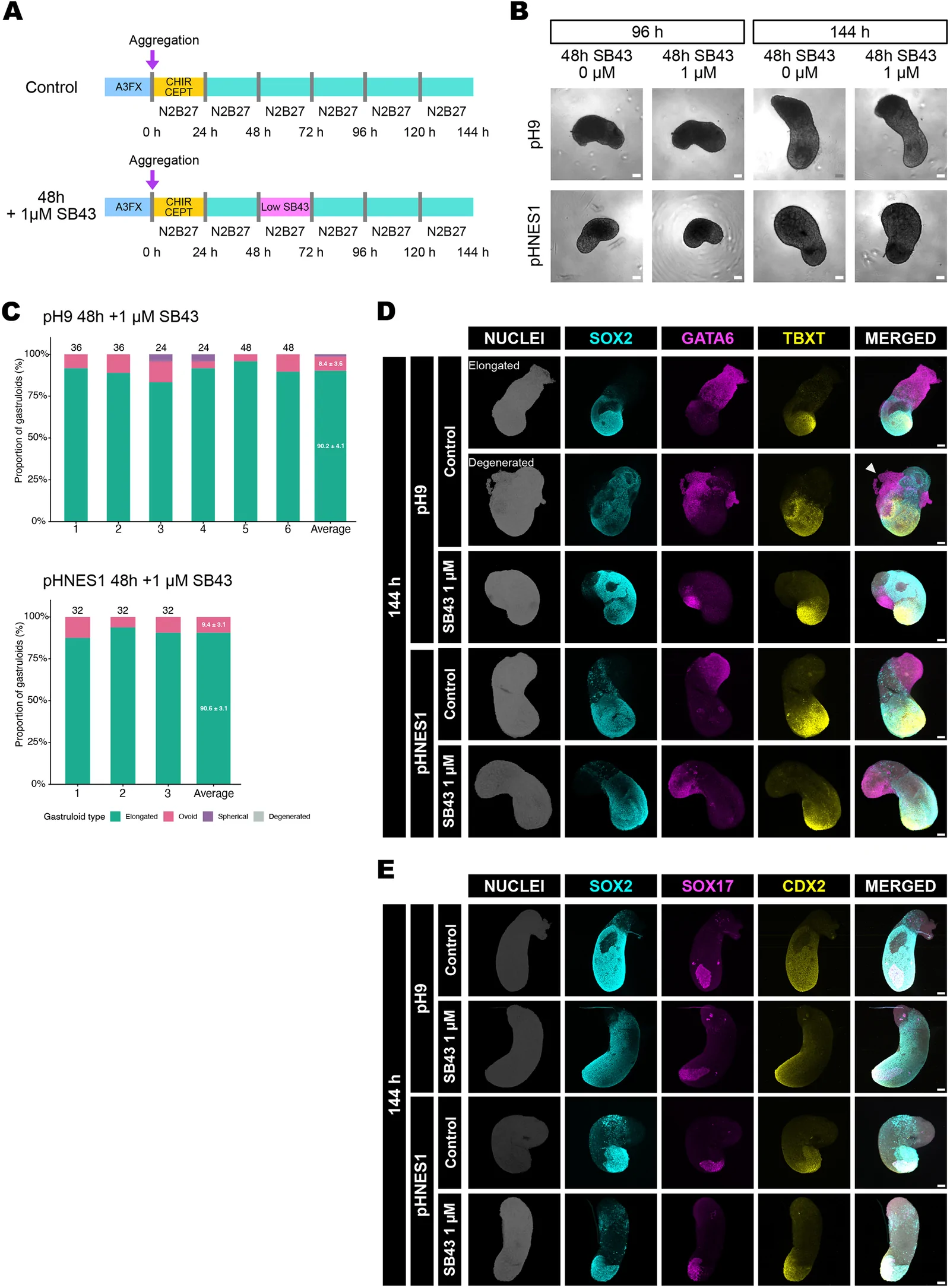
**Timely and low TGFβ inhibition improves gastruloid differentiation for H9 PSCs.** (A) Schematic of the protocol for making gastruloids without pre-CHIR treatment, but addition of CHIR and CEPT after aggregation. TGFβ inhibitor SB43 was added between 48 h and 72 h at low concentration (1 μM). (B) Representative images of gastruloid differentiation from pH9 and pHNES1 over the time course. (C) Proportion of gastruloids classified as elongated, ovoid, spherical or degenerated made from pH9 and pHNES1 hPSCs. The number of gastruloids analysed for each condition is indicated above the corresponding plot. (D) Immunofluorescence for SOX2, GATA6 and TBXT of 144 h gastruloids generated with or without SB43 at 48 h from A3FX-cultured pH9 and pHNES1 hPSCs. Arrowhead indicates broadly distributed GATA6^+^ cells. (E) Immunofluorescence for SOX2, SOX17, and CDX2 of 144 h gastruloids generated with or without SB43 at 48 h from A3FX-cultured pH9 and pHNES1 hPSCs. Scale bars: 100 μm.

To generate elongated gastruloids efficiently, with differentiation into all three germ layers, we optimized the timing of SB43 treatment (Fig. 4A). Administration of SB43 at a low concentration (1 or 2 μM) between 48 and 72 h after aggregation enabled efficient gastruloid elongation for both cell lines (Fig. 4B,C; Fig. S8A), while maintaining GATA6^+^ cardiac mesoderm and SOX17^+^ endoderm differentiation (Fig. 4D,E; Fig. S8B,C). Notably, degenerated pH9 gastruloids produced in the absence of SB43 exhibited a broad distribution of GATA6^+^ cells, rather than being restricted to the anterior region (Fig. 4D). In contrast, SB43 treatment produced gastruloids with a smoother morphology and anteriorly localized GATA6^+^ cells (Fig. 4D; Fig. S8B,C). Under these conditions, gastruloids continued to develop beyond 96 h and, by 144 h, SOX17^+^ cells could become organized into a structure resembling a gut tube (Fig. 4B,E).

To assess the applicability of this approach further, we applied the optimised N2B27+A3FX-based protocol to two additional human PSC lines: Shef6 (Aflatoonian et al., 2010), a primed human PSC line, and capacitated pHNES3, which was originally established under naïve conditions, coincident with the derivation of HNES1 (Guo et al., 2016) (Fig. S9). To derive gastruloids from Shef6 PSCs we initially used 1 μM SB43, which is optimal for pHNES1 and pH9. Although elongated Shef6 gastruloids could be generated, the efficiency was lower than for HNES1 or H9 gastruloid production. By increasing the concentration of SB43 to 2 μM, with no other changes to the protocol, Shef6 gastruloids could be produced efficiently (Fig. S9A,C). Shef6 gastruloids showed three lineage differentiation including SOX17^+^ endoderm and anterior-posterior patterning confirmed by GATA6, SOX2, TBXT and CDX2 expression and including structures resembling gut tubes (Fig. S10). By contrast, capacitated pHNES3 failed to generate elongated gastruloids under the same conditions (Fig. S9B,C). Given that HNES3 carries chromosomal abnormalities, including trisomy of chromosome 22 (Guo et al., 2016), this result suggests that line-specific genomic abnormalities may compromise developmental competence during gastruloid formation. In light of this finding, we propose that competence to generate gastruloids may serve as a simple method to assess the utility of candidate cell lines to be applied for creation of models of more advanced human organogenesis. In conclusion, by defining the key parameters governing human gastruloid formation, we have developed a simplified and robust protocol for efficient and consistent generation of elongated gastruloids from hPSCs (Fig. 4). We hope that the streamlined method for gastruloid production presented here will improve accessibility and reproducibility, providing a controlled platform to investigate fundamental principles of early human development.

## MATERIALS AND METHODS

### Ethics statement

The human cell lines used in this study comprise: H9, one of the first PSC lines to be derived directly from blastocysts in the USA (Thomson et al., 1998), which has been widely used by the stem cell and developmental biology community; Shef6, derived directly from blastocysts using the microdrop culture system in the UK (Aflatoonian et al., 2010); and HNES1 and HNES3 (Guo et al., 2016), derived in UK from disaggregated isolated ICM from D6 blastocysts, donated to Human Fertility and Embryology Authority (HFEA) licence R0178, with informed consent from patients undergoing assisted conception procedures, for purposes including derivation of PSC lines stipulated in the licence application and patient information document. The gastruloids generated from these cells lack extra-embryonic tissues, anterior structures and circulating blood; they therefore pose little risk of acquiring organised brain tissue or sentience and consequently require no specific ethical approval.

### hPSC culture

H9, Shef6, primed HNES1 and primed HNES3 hPSCs were used in this study. Naïve HNES1 and HNES3 were capacitated to the primed state according to the previously reported protocol (Rostovskaya et al., 2019). All hPSCs were cultured in N2B27 supplemented with 3 ng/ml or 20 ng/ml activin A (Qkine), 12.5 ng/ml FGF2 (Qkine) and 2 μM XAV939 (Tocris) on 1:100 diluted Geltrex (Gibco)-coated plates at 37°C, 7% CO_2_, 5% O_2_. hPSCs were passaged every 4 days using Accutase (BioLegend). ROCK inhibitor Y-27632 (Tocris) was used at 10 μM when passaging hPSCs. The compositions of the N2B27 and AFX media are provided in Table S1.

### Conventional human gastruloid induction

Conventional human gastruloids were induced according to previous reports (Moris et al., 2020) with modifications. hPSCs were plated at 2×10^4^, 4×10^4^, 6×10^4^ or 8×10^4^ cells per well onto 12-well plates coated with 1:100 diluted Geltrex in DMEM/F-12 and cultured in N2B27 containing either A3FX or A20FX plus 10 μM Y-27632. Between day 1 and day 3, the medium was changed to N2B27 with AF3X or A20FX. On day 4, the medium was replaced with N2B27 supplemented with 3 ng/ml or 20 ng/ml activin A, 10 ng/ml FGF2 and 4 μM CHIR99021. After 24 h, cells were dissociated with Accutase. Then, 500 cells were plated into round bottom 96-well plate with 40 μl N2B27 with 4 μM CHIR99021, CEPT cocktail (Chen et al., 2021; Ryu et al., 2024; Tristan et al., 2023). After 24 h, 150 μl N2B27 was added to each well. From 48 h, 150 μl of medium was removed and 150 μl of fresh N2B27 was added to each well by multi-channel pipette. Gastruloids were cultured until 96 h.

### Gastruloid induction with direct aggregation

hPSCs were cultured in N2B27 with A3FX on 1:100 diluted Geltrex-coated plate for 4 days until they formed compacted colonies with ∼80% confluency. Cells were dissociated with Accutase. Then, 500 or 750 cells were plated into round bottom 96-well plates containing 40 μl N2B27 with 4-5 μM CHIR99021 and CEPT cocktail. At 24 h or 48 h, 150 μl of N2B27 medium with or without SB431542 (1 μM, 2 μM or 10 μM; Tocris) was added to each well. From 48 h onward, 150 μl of medium was removed from each well and replaced with 150 μl of fresh N2B27 using a multichannel pipette. Gastruloids were cultured until 144 h.

### Immunofluorescence

For immunofluorescence of hPSCs in 2D, cells were cultured on plastic cover slips coated with 1:100 diluted Geltrex. Cells were fixed at the indicated time points in 4% paraformaldehyde (PFA) for 15 min at room temperature (RT), followed by washing in PBS. Cells were permeabilized in 0.1% Triton X-100 in PBS for 10 min at RT. Blocking reaction was carried out with 5% donkey serum, 5% bovine serum albumin (BSA) and 0.1% Triton X-100 in PBS for 1 h at RT. Primary antibodies (Table S2) were diluted in blocking solution and incubated overnight at 4°C. After washing cells in 0.1% Tween 20 in PBS, they were incubated with Alexa Fluor-conjugated secondary antibodies (Thermo Fisher Scientific) diluted 1:500 in blocking solution for 1 h at RT. Nuclear staining was carried out with Hoechst 33342 (Thermo Fisher Scientific).

Gastruloids were collected from each stage and fixed in 4% PFA overnight at 4°C, followed by washing in PBS. Gastruloids were permeabilized in 0.5% Triton X-100 in PBS for 15 min and blocked with 5% donkey serum, 5% BSA and 0.1% Tween20 in PBS overnight at 4°C. Primary antibodies (Table S2) were diluted in blocking solution and incubated overnight at 4°C. After washing in 0.1% Tween 20 in PBS, gastruloids were incubated with Alexa Fluor-conjugated secondary antibodies (Thermo Fisher Scientific) for 2 h at RT. Nuclear staining was carried out with Hoechst 33342 (Thermo Fisher Scientific).

### Quantification of fluorescence intensity profiles along the gastruloid A-P axis

Fluorescence intensity profiles along the gastruloid anterior-posterior (A-P) axis were quantified using a custom pipeline adapted from the contour-based approach described previously (Merle et al., 2024). Maximum-projection images were segmented using the nuclear channel to generate a gastruloid mask and contour. A smooth midline was estimated along the major body axis, and transverse sections were sampled at evenly spaced positions along the normalized A-P coordinate. Marker-channel intensities were measured across each section, averaged, and assigned to a normalized position ‘t’ from 0 to 1. The orientation of the axis was assigned using marker expression, with t=0 corresponding to the anterior marker-enriched side and t=1 corresponding to the posterior marker-enriched side.

For final quantification, background-subtracted mean intensities were used. Per-sample min-max normalization was used only for quality-control visualisation but not for condition-level analysis. Instead, all background-subtracted profiles from the same experiment, cell line and marker were pooled across conditions and normalized to a common 99th-percentile intensity scale. Condition-level profiles were plotted as the mean normalized intensity±s.e.m. across individual gastruloids.

### Imaging and image analysis for 2D cultures

Brightfield images for gastruloids were acquired using the EVOS M7000 imaging system (Invitrogen). Fluorescent images for gastruloids were acquired using a Stellaris 8 confocal microscope (Leica) and processed using ImageJ/Fiji. Nuclear segmentation was performed using Cellpose3 (Stringer et al., 2021). The resulting regions of interest (ROIs) were transferred to each channel in Fiji, and the mean intensity of each cell was quantified using the ‘Measure’ function.

To quantify marker co-expression, mean fluorescence intensities for TBXT, FOXA2 and SOX2 were normalized to the nuclear channel intensity (Hoechst 33342) for each segmented cell. Cells were classified into eight mutually exclusive categories based on normalized intensity ratios exceeding a threshold of 2.5 for each marker: Triple+ (TBXT^+^/LEF1^+^/SOX2^+^), double-positive populations (TBXT^+^/LEF1^+^, TBXT^+^/SOX2^+^, LEF1^+^/SOX2^+^), single-positive populations (TBXT^+^, LEF1^+^, SOX2^+^) and Negative. The fraction of cells in each category was calculated as a percentage of the total segmented cells per condition. The expression level of each gene was normalized to the corresponding Hoechst channel intensity and plotted. To compare expression levels between the colony centre and periphery, intensity profiles across colonies were quantified using the ‘Plot Profile’ function in Fiji. The resulting intensity for each channel was then visualized as a line plot.

### Imaging and image analysis for gastruloids

Brightfield images for gastruloids were acquired using the EVOS M7000 imaging system (Invitrogen). Fluorescent images for gastruloids were acquired using a Stellaris 8 confocal microscope (Leica) and processed with Fiji.

### Morphological quantification of gastruloids

The proportions of gastruloid morphologies (spherical, ovoid, elongated and degenerated) were quantified across multiple independent biological replicates using a custom, in-house image-analysis pipeline derived from previously described approaches (Girgin et al., 2021). Briefly, brightfield images were converted to single-channel, 8-bit TIFF files using Fiji (ImageJ). Images were inverted and background-corrected using a rolling-ball subtraction algorithm (radius=200 pixels), followed by Gaussian smoothing (σ=5.0) before segmentation. Binary masks were generated using automatic thresholding (Otsu et al., 2014), followed by morphological opening and hole filling to remove noise and internal artefacts.

Morphological measurements were extracted using a maximum inscribed circle-fitting strategy. For each segmented gastruloid, a series of maximum inscribed circles (minimum diameter=4 pixels) was fitted along the object, and the centroids of these circles were connected to define a continuous midline. Axial length was calculated as the cumulative length of this path. An EI was defined as the ratio of axial length to the diameter of the largest inscribed circle. Additional quantitative features, including circularity and roundness, were extracted from object contours. Based on these shape descriptors, gastruloids were automatically classified into four morphological categories: spherical, defined by an axial length <230 pixels; elongated, defined by EI≥1.8; ovoid, defined as samples not meeting the elongated criterion but exhibiting either a smooth surface (circularity>0.80) or intermediate elongation (1.5≤EI<1.8); and degenerated, defined by a rough surface (circularity≤0.80) combined with low elongation (EI<1.5). Images were quality-controlled to exclude empty wells or samples containing debris that interfered with shape quantification, based on area and circularity outliers, and confirmed by manual inspection. This analysis pipeline was used to assess the reproducibility and morphological heterogeneity of human gastruloid formation across experimental conditions.

## Supplementary Material



10.1242/develop.205719_sup1 Supplementary information
