# Supplementary material for: Simplified in vitro generation of human gastruloids for modelling early development
Source: Development. 2026 Oct 23;153(20):dev205719. doi: 10.1242/dev.205719 (PMC13723424; doi:10.1242/dev.205719)
Supplement: Supplementary file 1 [file develop-153-205719-s1.pdf]

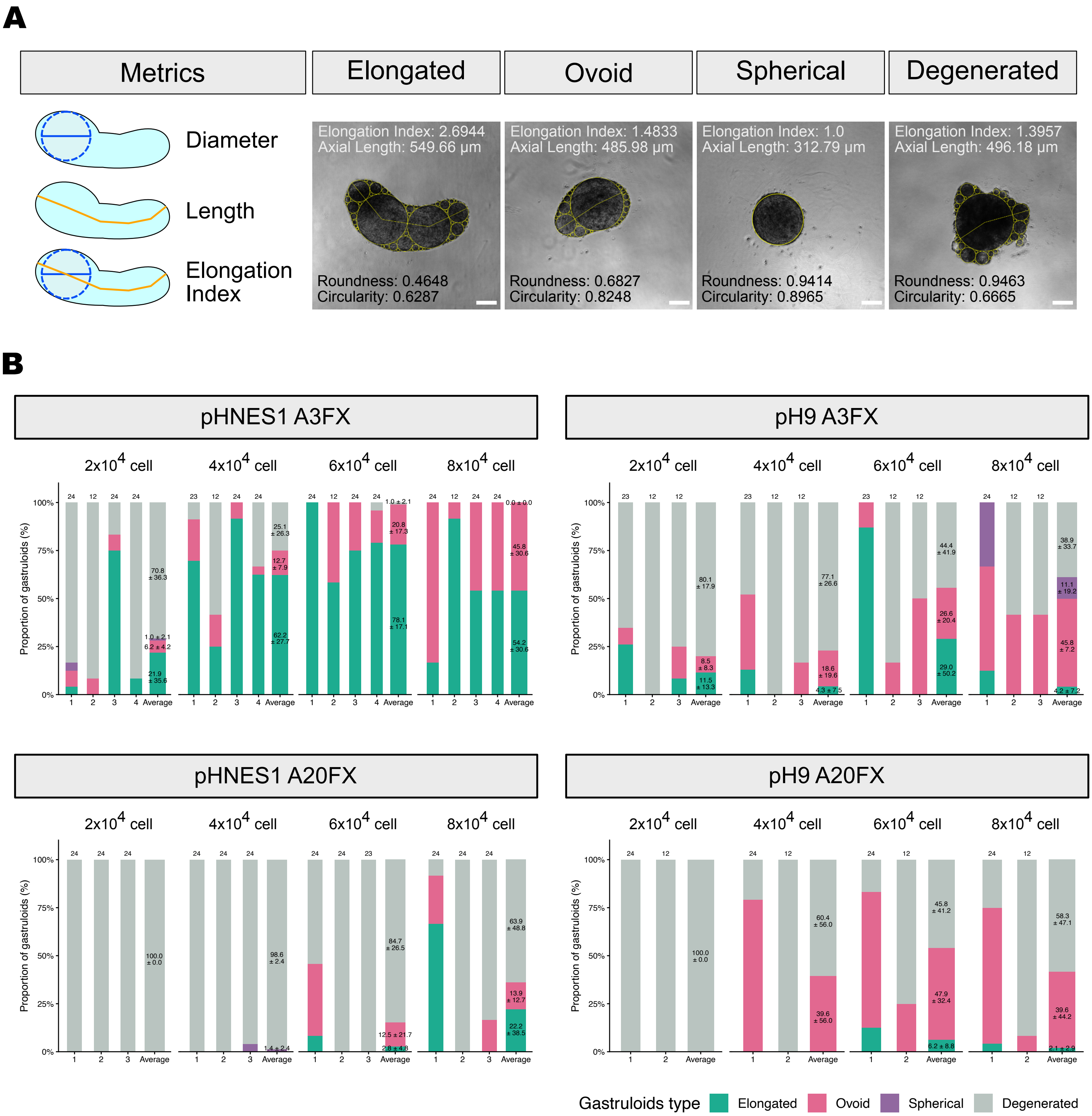

Development • Supplementary information

Fig. S1. Morphological characterisation and proportions of gastruloid structures generated from pHNES and pH9 pluripotent stem cells.

(A) Schematics to illustrate the maximum inscribed circle–fitting approach used to extract morphological features. For each gastruloid, inscribed circles were fitted along the object to define a midline for axial length measurement and to determine the diameter of the largest circle used to calculate the Elongation Index. Additional shape descriptors, including circularity and roundness, were extracted from object contours and used to classify gastruloids into four morphological categories: elongated, ovoid, spherical, and degenerated. Representative images of each category and the corresponding quantitative metrics are shown. Scale bar = 100 μm. (B) Proportion of elongated gastruloids generated from pHNES1 and pH9 cells cultured in A3FX or A20FX conditions at 96 h, quantified using an automated morphometric elongation metric. Left: proportions from individual biological replicates; right: mean proportions with percentages and mean ± SD values indicated.

Development • Supplementary information

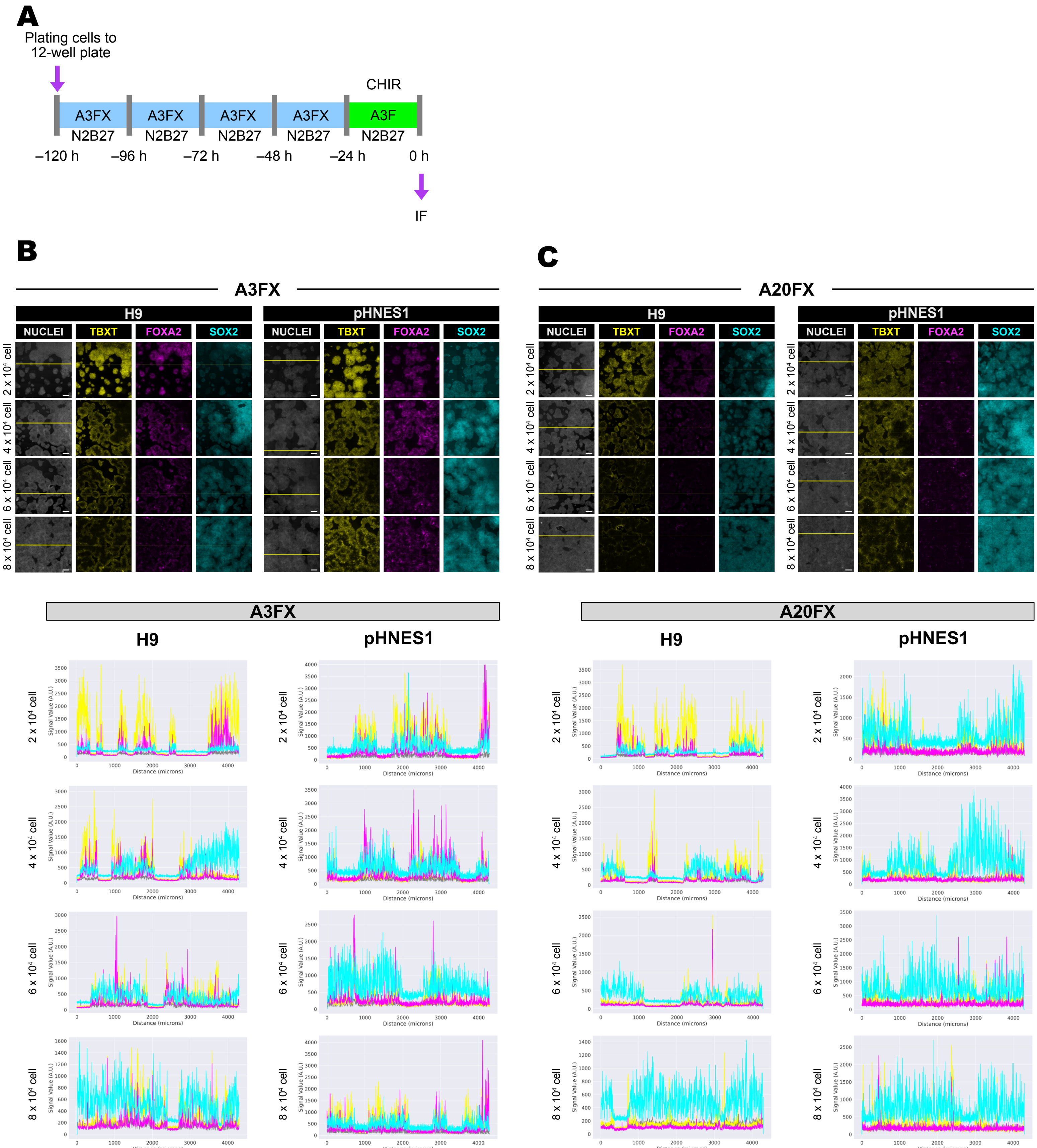

Development • Supplementary information

Fig. S2. Immunofluorescence quantification of lineage markers TBXT, FOXA2 and SOX2 of H9 or pHNES1 cells cultured at various densities in low Activin A. (A) Schematic for the culture and pre-CHIR treatment of hPSCs. (B, C) Immunofluorescence for TBXT, FOXA2 and SOX2 in pre-CHIR treated pH9 and pHNES1 from A3FX (B) or A20FX (C). hPSCs were plated at the indicated cell number in 12-well plate and cultured for 4 days in A3FX or A20FX; pre-CHIR treatment was performed with 4 μM CHIR/A3F or A20F for 24 h. The image was generated by stitching together a 5x5 tile scan. Scale bar = 500 μm. To compare expression at the colony centre and periphery, signal intensities for TBXT, FOXA2, and SOX2 were measured along the yellow line (nuclei channel) using Plot Profile and visualized as a line plot.

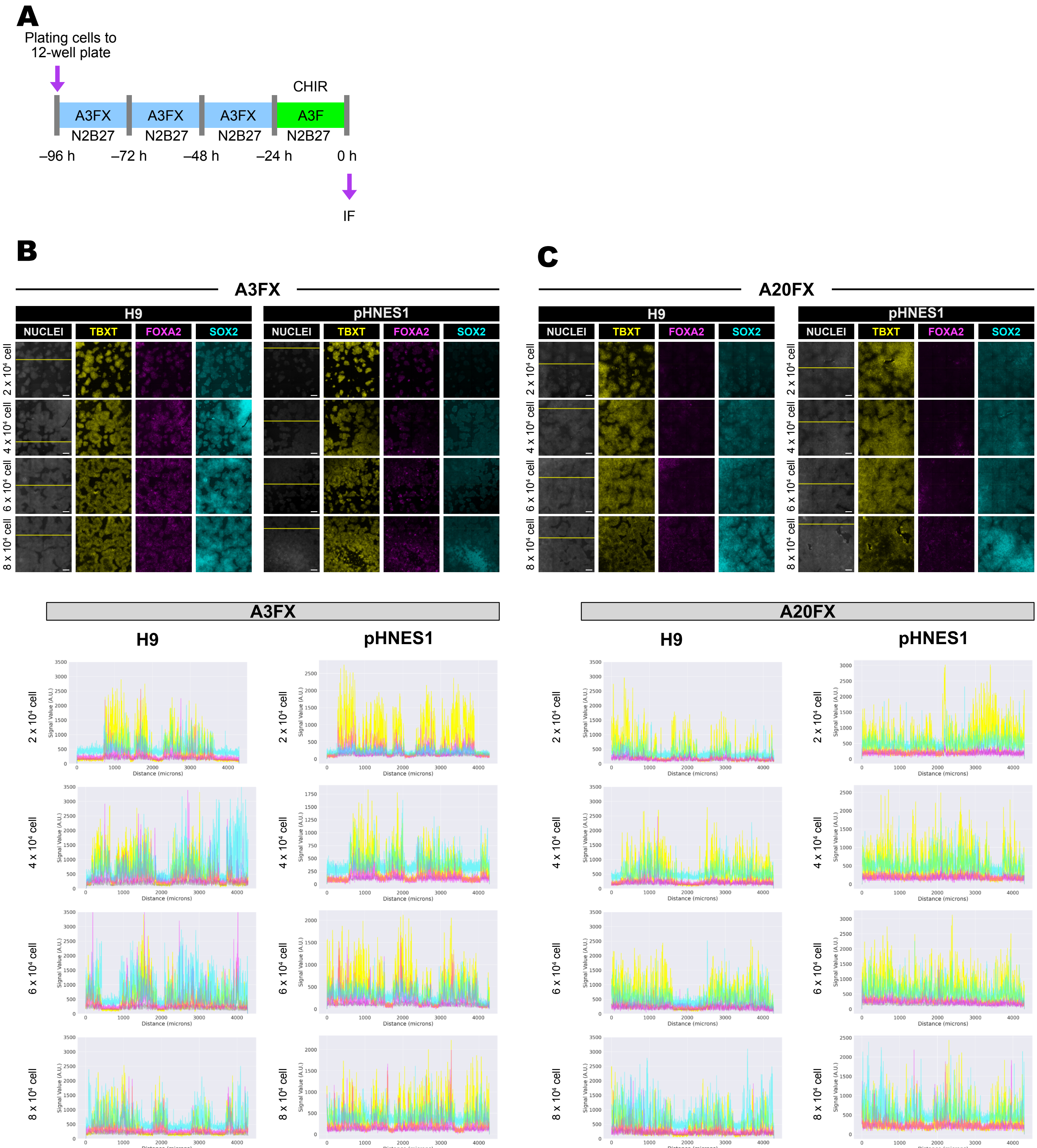
A
Plating cells to
12-well plate
CHIR
A3FX
A3FX
A3FX
A3F
N2B27
N2B27
N2B27
N2B27
–96 h
–72 h
–48 h
–24 h
0 h
IF
B
A3FX
H9
pHNES1
NUCLEI
TBXT
FOXA2
SOX2
2 x 10⁴ cell
4 x 10⁴ cell
6 x 10⁴ cell
8 x 10⁴ cell
C
A20FX
H9
pHNES1
NUCLEI
TBXT
FOXA2
SOX2
A3FX
H9
pHNES1
A20FX
H9
pHNES1
Signal Value (A.U.)
Distance (microns)

Fig. S3. Immunofluorescence quantification of lineage markers TBXT, FOXA2 and SOX2 of H9 or pHNES1 cells pre-cultured in Chir and low Activin A.
(A) Schematic for the culture and pre-CHIR treatment of hPSCs. (B, C) Immunofluorescence for TBXT, FOXA2, and SOX2 in pre-CHIR treated pH9 and pHNES1 PSCs from A3FX (A) or A20FX (B). hPSCs were plated at the indicated cell number in 12-well plate and cultured for 3 days in A3FX or A20FX, and pre-CHIR treatment was performed with 4 µM CHIR/A3F or A20F for 24 h. The image was generated by stitching together a 5x5 tile scan. Scale bar = 500 µm. To compare expression at the colony centre and periphery, signal intensities for TBXT, FOXA2, and SOX2 were measured along the yellow line (nuclei channel) using Plot Profile and visualized as a line plot.

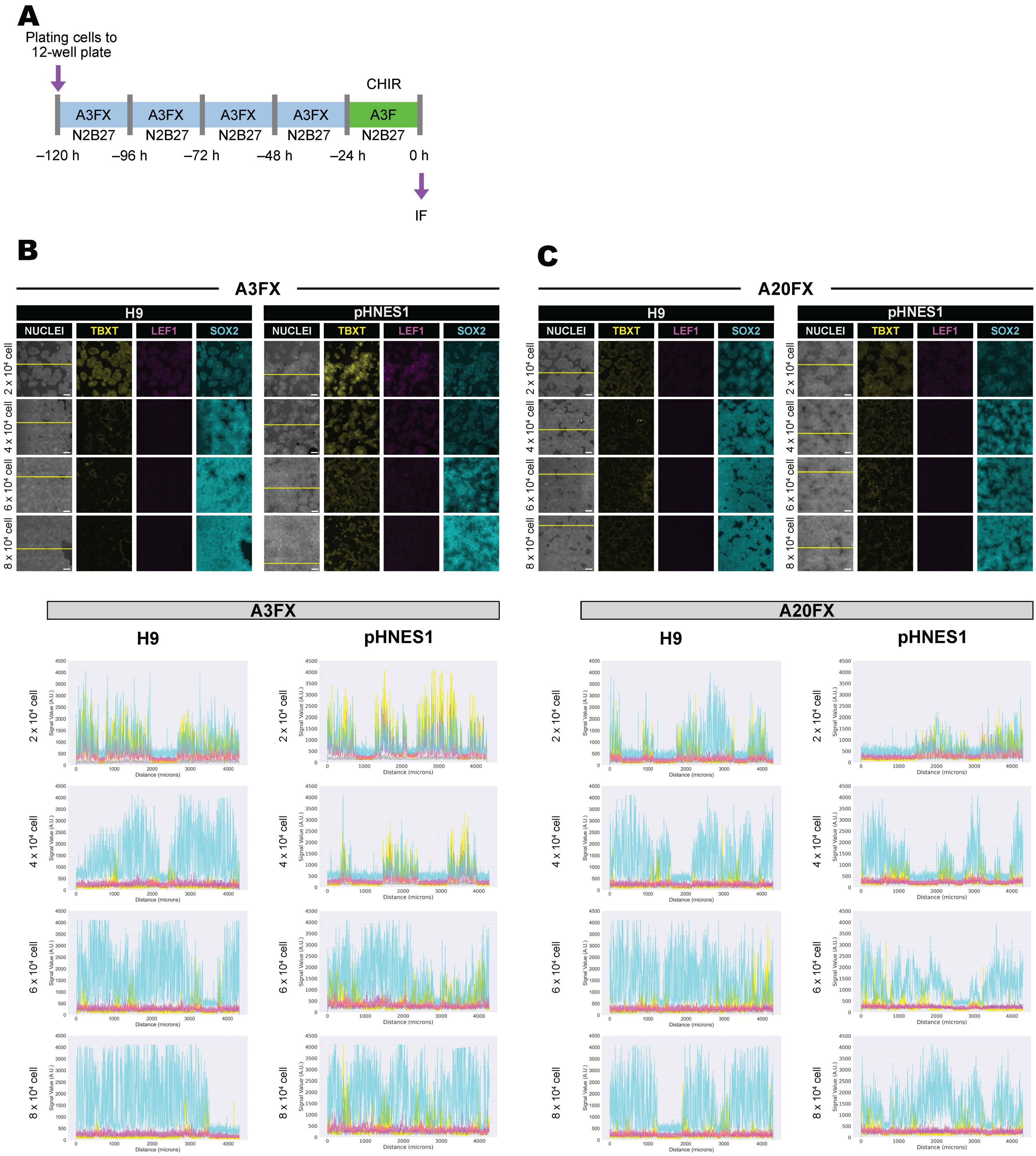

Fig. S4. Immunofluorescence quantification of lineage markers TBXT, LEF1 and SOX2 of H9 or pHNES1 cells at various densities in low Activin A.

(A) Schematic for the culture and pre-CHIR treatment of hPSCs. (B, C) Immunofluorescence for TBXT, LEF1, and SOX2 in pre-CHIR treated pH9 and pHNES1 hPSCs from A3FX (B) or A20FX (C). hPSCs were plated at the indicated cell number in 12-well plate and cultured for 4 days in A3FX or A20FX, and pre-CHIR treatment was performed in 4 µM CHIR/A3F or A20F for 24 h. The image was generated by stitching together a 5x5 tile scan. Scale bar = 500 µm. To compare expression at the colony centre and periphery, signal intensities for TBXT, LEF1, and SOX2 were measured along the yellow line (nuclei channel) using Plot Profile and visualized as a line plot.

Development • Supplementary information

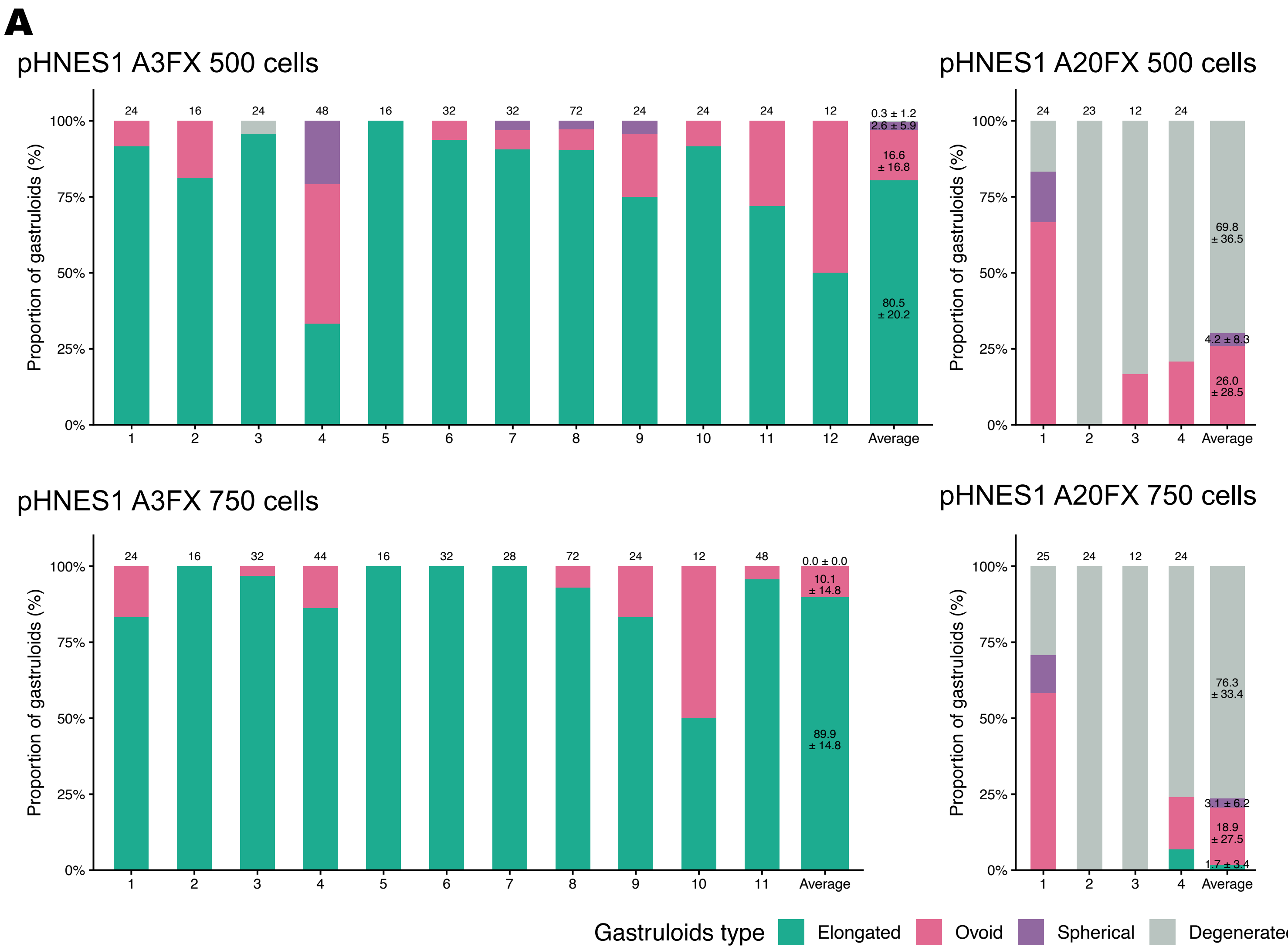

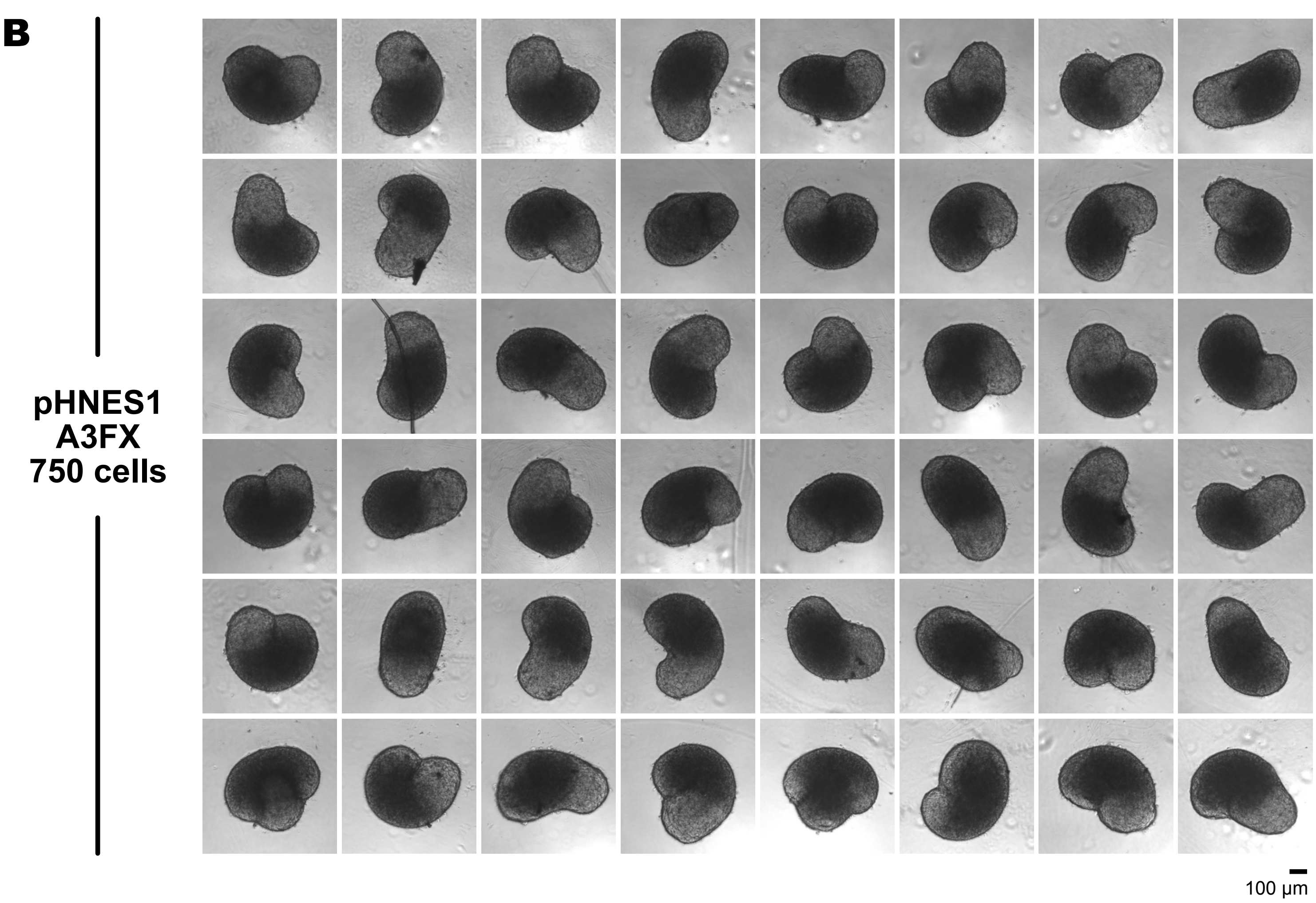

Development • Supplementary information

Fig. S5. Efficient elongation of gastruloids from aggregation of 750 pHNES1 cells in A3FX.

(A) Proportion of elongated gastruloids generated by the direct aggregation protocol from pHNES1 cultured in A3FX or A20FX conditions at 96 h, quantified using an automated morphometric elongation metric. Left: proportions from individual biological replicates. Right: mean proportions with percentages and mean ± SD values indicated. (B) Bright field images of gastruloids generated from pHNES1 in A3FX using direct aggregation protocol. Scale bar = 100 μm.

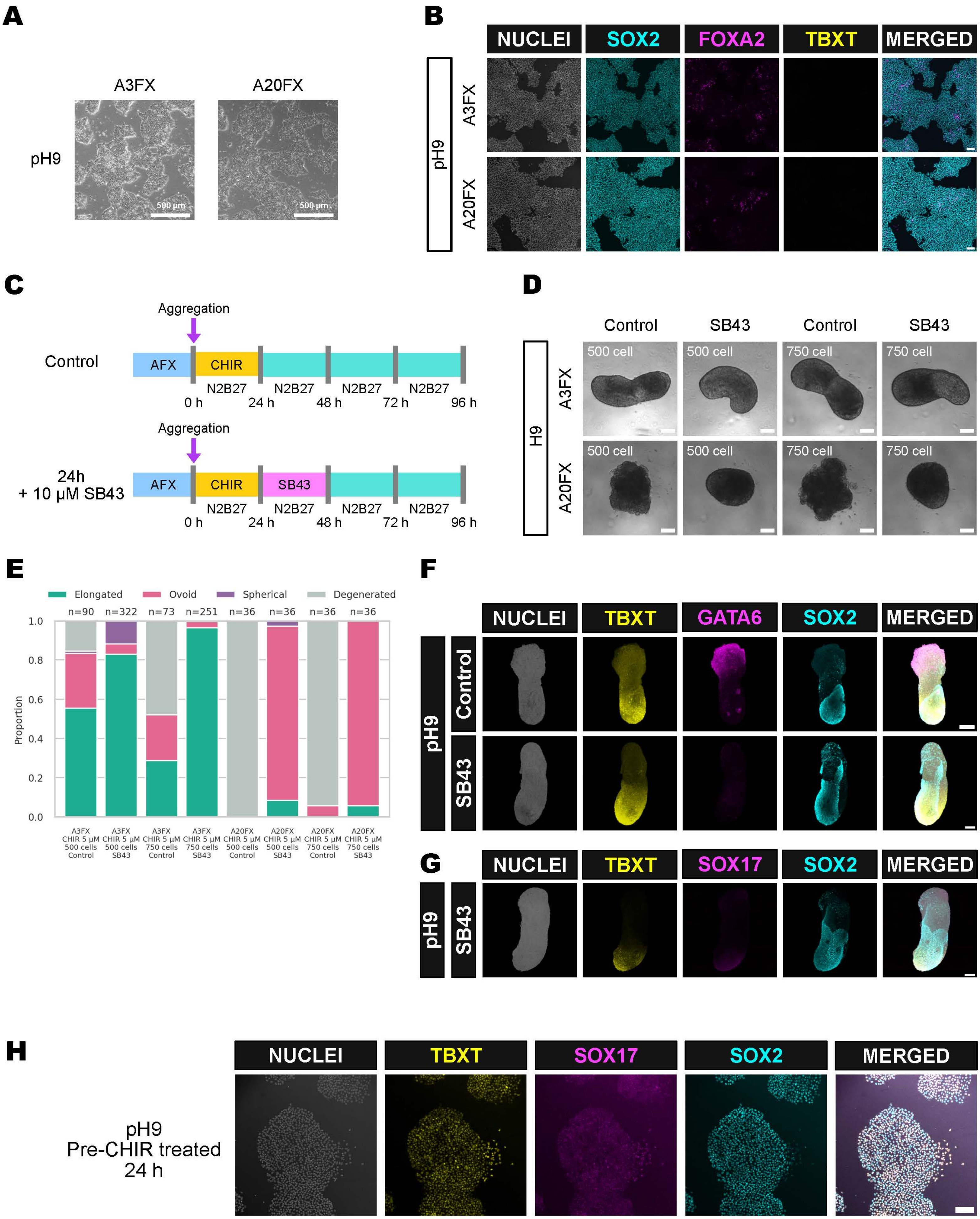

Development • Supplementary information

Fig. S6. Enhanced gastruloid formation from pH9 cells cultured in presence of SB43.

(A) Representative bright field images for A3FX and A20FX culture pH9 hPSCs before making gastruloids. Scale bar = 500 µm. (B) Immunofluorescence for SOX2, FOXA2, and TBXT in A3FX and A20FX cultured pH9 hPSCs. Scale bar = 100 µm. (C) Schematic for the protocol for making gastruloids without pre-CHIR treatment. SB43 was added between 24 h and 48 h at 10 µM. (D) Representative images of gastruloid differentiation with indicated condition. Scale bar = 100 µm. (E) Proportion of elongated, ovoid, spherical, and degenerated morphology from pH9 hPSCs generated from the indicated condition. The number of gastruloids (n) for each condition is indicated above the corresponding plot. The plots represent data pooled from 5 (A3FX control), 10 (A3FX SB43), 3 (A20FX control), and 3 (A20FX SB43) independent experiments. (F) Immunofluorescence for TBXT, GATA6 and SOX2 of gastruloids generated with or without SB43 from A3FX cultured pH9 hPSCs. Scale bar = 100 µm. (G) Immunofluorescence for TBXT, SOX17 and SOX2 of gastruloids generated with SB43 from A3FX cultured pH9 hPSCs. Scale bar = 100 µm. (H) Immunofluorescence for TBXT, SOX17 and SOX2 of pre-CHIR treated pH9 hPSCs. Scale bar = 100 µm.

Development • Supplementary information

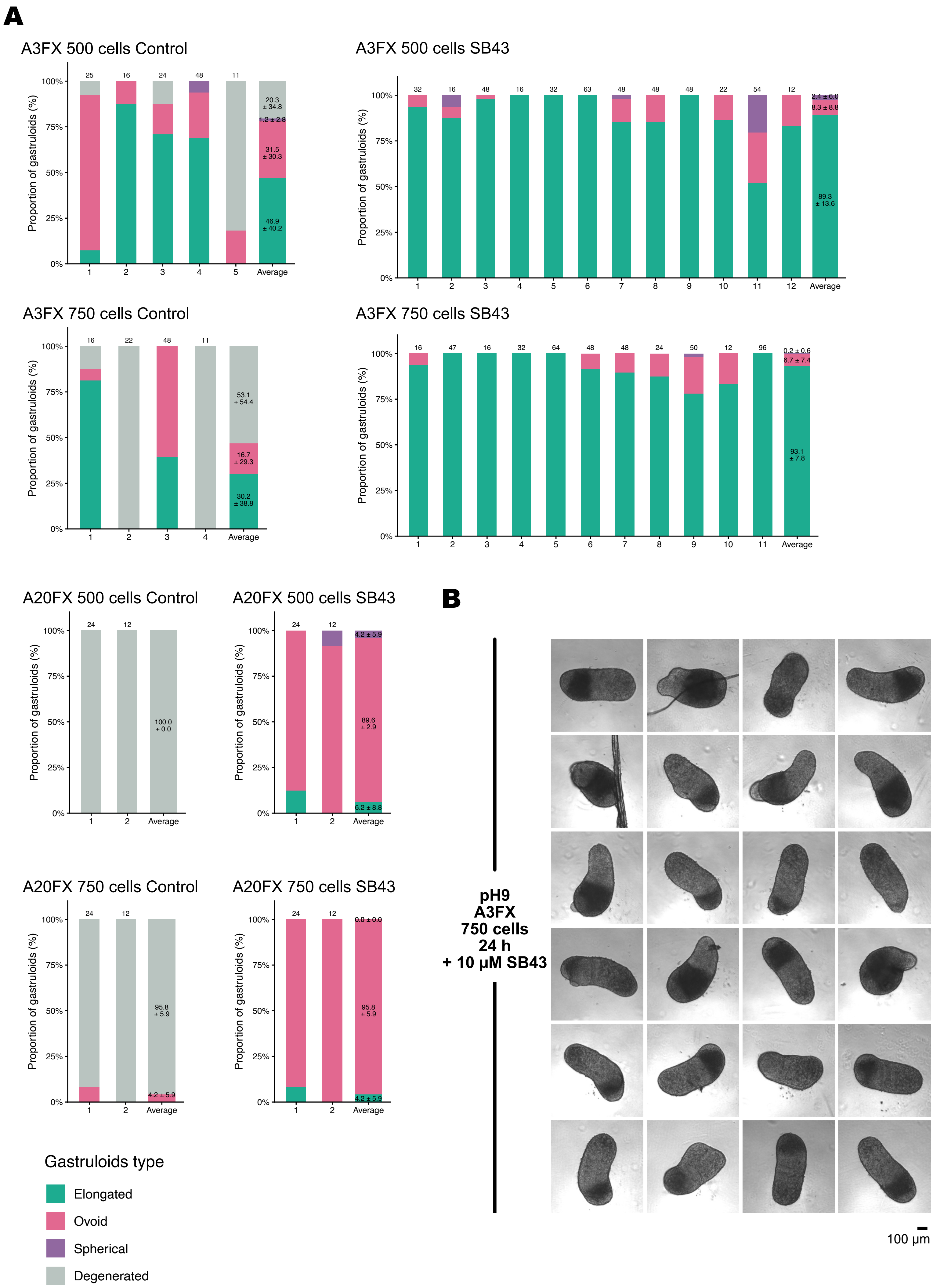

Development • Supplementary information

Fig. S7. Efficient elongation of gastruloids by direct aggregation of 750 pH9 cells.

(A) Proportion of elongated gastruloids generated by the direct aggregation protocol from pH9 cultured in A3FX or A20FX conditions at 96 h, quantified using an automated morphometric elongation metric. Control (right), without SB43; experimental (left): SB43 added between 24 h and 48 h after aggregation. Left bars: proportions from individual biological replicates. Right bar: mean proportions with percentages and mean ± SD values indicated. (B) Representative images of 96 h gastruloids generated by the direct aggregation protocol from pH9 hPSCs cultured in A3FX. SB43 was added between 24 h and 48 h at 10 μM. Scale bar = 100 μm.

Development • Supplementary information

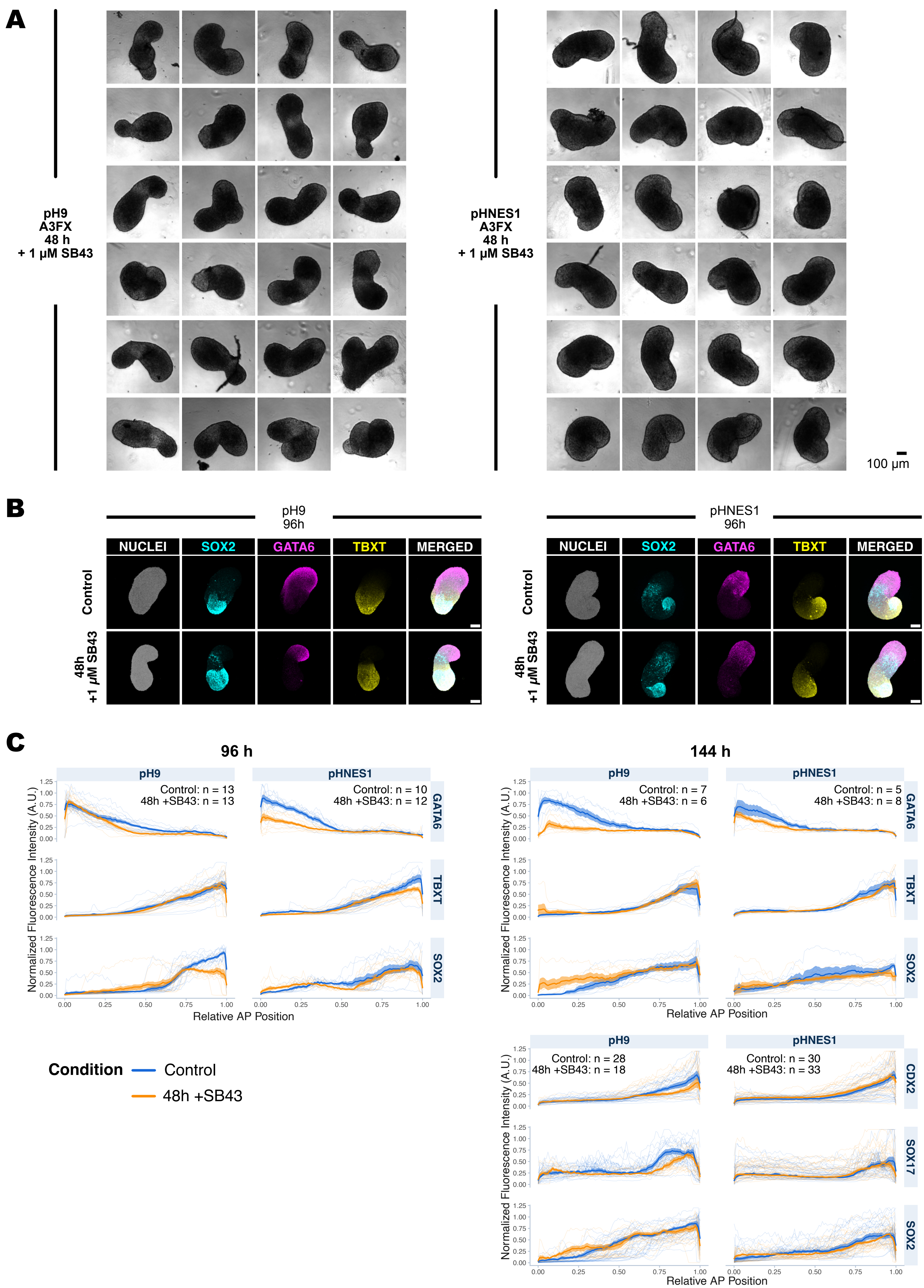

Development • Supplementary information

Fig. S8. Immunofluorescence for SOX2, GATA4 and TBXT of pH9 and pHNES1 cells cultured with or without SB43.

(A) Representative images of 96 h gastruloids generated by the direct aggregation protocol from pH9 and pHNES1 hPSCs cultured in A3FX. SB43 was added between 48h and 72 h at low concentration (1 μM). Scale bar = 100 μm. (B) Immunofluorescence for SOX2, GATA6, and TBXT of 96 h gastruloids generated with or without 1 μM SB43 at 48 h from A3FX cultured pH9 and pHNES1 hPSCs. Scale bar = 100 μm. (C) Relative axial fluorescence intensity profiles of GATA6, TBXT and SOX2 in 96 h and 144 h gastruloids (left and top right) and CDX2, SOX17, and SOX2 in 144 h gastruloids (bottom right) along the anteroposterior axis of human gastruloids. Profiles are shown for pH9 and pHNES1 gastruloids under control and 48 h SB43-treated conditions, as determined using axial fluorescence patterning quantification. The x-axis indicates the normalized anteroposterior position, with 0 corresponding to the anterior pole and 1 to the posterior pole. Thin lines represent individual gastruloids; thick lines show the condition mean, and shaded regions indicate ± SEM. The number of gastruloids analysed for each condition is shown in the plot.

Development • Supplementary information

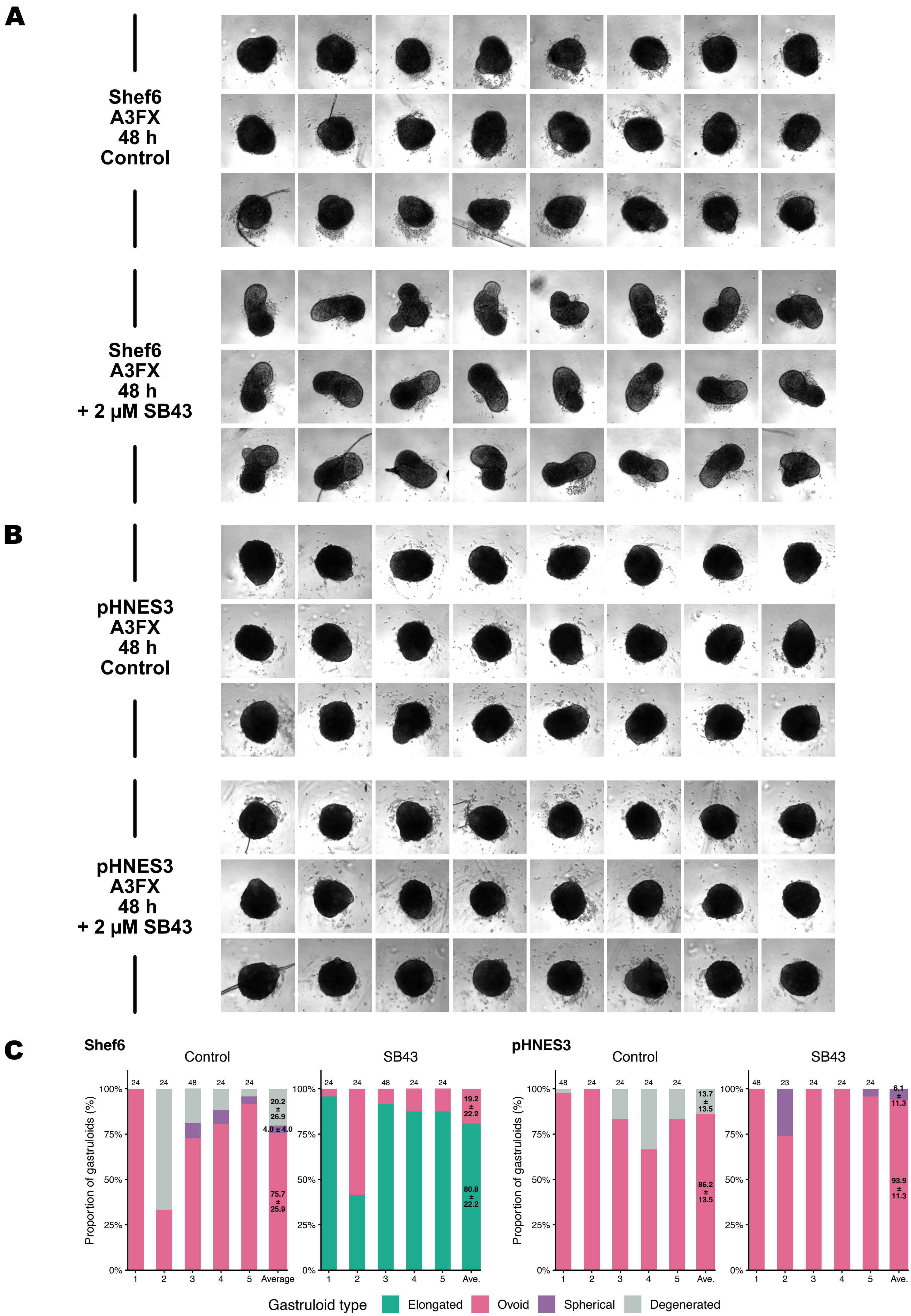
A
Shef6
A3FX
48 h
Control
Shef6
A3FX
48 h
+ 2 µM SB43
B
pHNES3
A3FX
48 h
Control
pHNES3
A3FX
48 h
+ 2 µM SB43
C
Shef6
Control
SB43
pHNES3
Control
SB43
Proportion of gastruloids (%)
100%
75%
50%
25%
0%
1
2
3
4
5
Average
Ave.
24
48
23
20.2 ± 26.9
4.0 ± 4.0
75.7 ± 25.9
19.2 ± 22.2
80.8 ± 22.2
13.7 ± 13.5
86.2 ± 13.5
6.1 ± 11.3
93.9 ± 11.3
Gastruloid type
Elongated
Ovoid
Spherical
Degenerated

Fig. S9. Efficient elongation of gastruloids confirmed using Shef6 cells cultured with SB43.

(A, B) Representative images of 96 h gastruloids generated by the direct aggregation protocol from Shef6 and pHNES3 hPSCs cultured in A3FX. SB43 was added between 48 and 72 h at low concentration (2 μM). (C) Proportion of elongated gastruloids generated by the direct aggregation protocol from Shef6 (left) and pHNES3 (right) cultured in A3FX conditions at 96 h, quantified using an automated morphometric elongation metric. Control, without SB43; 2μM SB43 added between 48 h and 72 h after aggregation. Left 5 bars: proportions from individual biological replicates; right bars: mean proportions with percentages and mean ± SD values indicated.

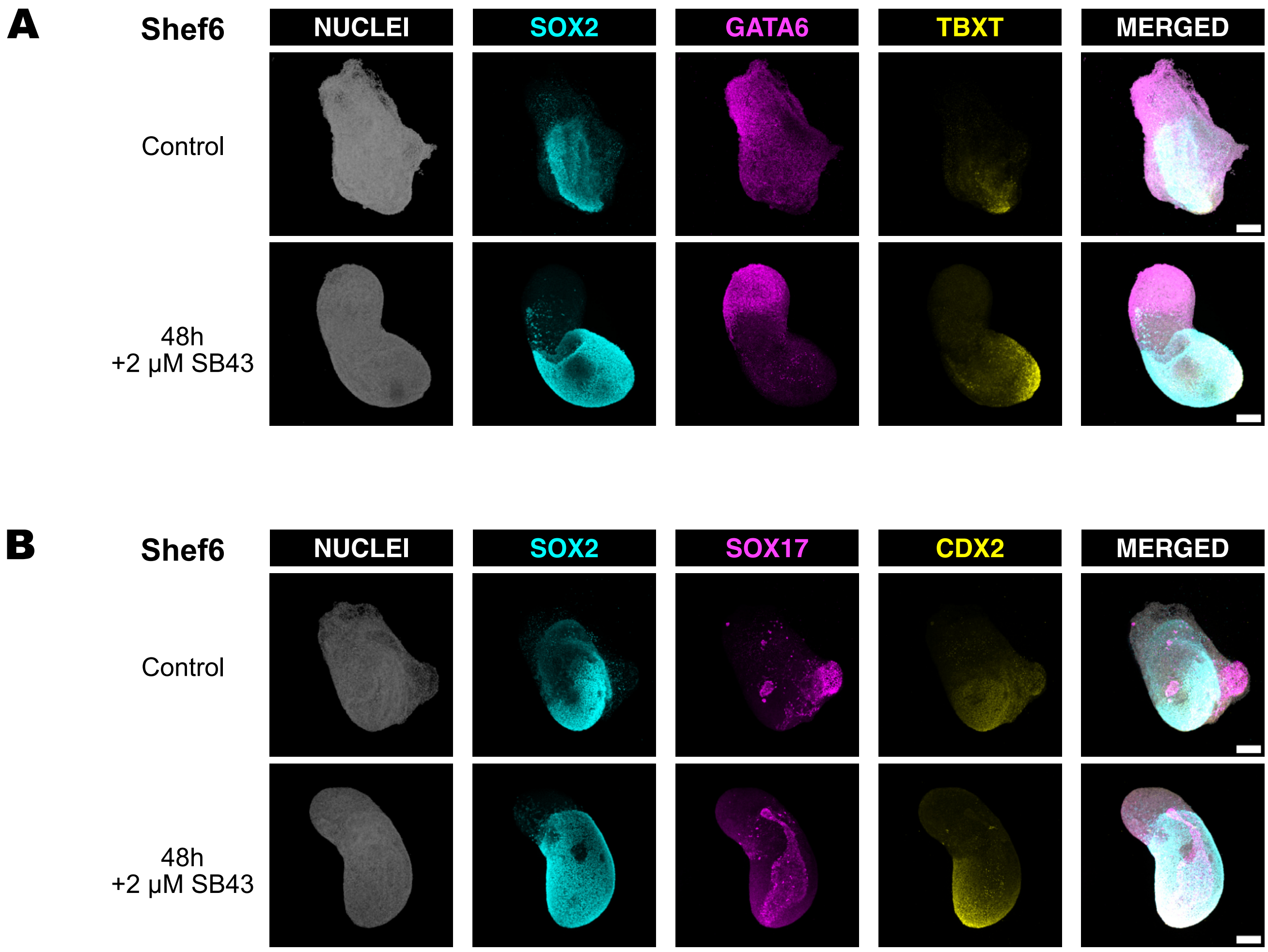

Fig. S10. Immunofluorescence for SOX2, GATA6, SOX17, TBXT and CDX2 showing efficient elongation of Shef6 cells in presence of SB43.

(A) Immunofluorescence for SOX2, GATA6, and TBXT of 144 h gastruloids generated with or without SB43 from A3FX cultured Shef6 hPSCs. For SB43 condition, 2 μM SB43 was added between 48h and 72h after aggregation. Scale bar = 100 μm. (B) Immunofluorescence for SOX2, SOX17, and CDX2 of 144 h gastruloids generated with or without SB43 from A3FX cultured Shef6 hPSCs. With SB43 condition, 2 μM SB43 was added between 48h and 72h after aggregation. Scale bar = 100 μm.

Development • Supplementary information

Table S1. Media composition

N2B27 medium

Component	Source (Cat#)	Volume
DMEM F-12	Gibco (21331-046)	98 mL
Neurobasal	Gibco (21103-049)	98 mL
B-27™ Supplement (50X), serum free	Gibco (17504044)	2 mL
Neuro2 supplement	Merck (SCM012)	0.5 mL
L-Glutamine (200 mM)	Gibco (25030081)	2 mL
MEM Non-Essential Amino Acids Solution (100X)	Gibco (111400)	2 mL
Sodium Pyruvate (100 mM)	Gibco (11360070)	2 mL
2-Mercaptoethanol (50 mM)	Gibco (31350010)	200 µL
total		200 mL

A3FX medium

Component	Source (Cat#)	Stock conc.	Final conc.	Volume
N2B27			1X	50 mL
Activin A	Qkine (Qk001)	50 µg/mL	3 ng/mL	3 µL
FGF2	Qkine (Qk052)	50 µg/mL	12.5 ng/mL	12.5 µL
XAV939	Tocris (3748)	20 mM	2 µM	5 µL
total				50 mL

A20FX medium

Component	Source (Cat#)	Stock conc.	Final conc.	Volume
N2B27			1X	50 mL
Activin A	Qkine (Qk001)	50 µg/mL	20 ng/mL	20 µL
FGF2	Qkine (Qk052)	50 µg/mL	12.5 ng/mL	12.5 µL
XAV939	Tocris (3748)	20 mM	2 µM	5 µL
total				50 mL

Table S2. List of primary and secondary antibodies with recommended concentrations

Primary antibodies	Source	Cat#	Dilution
Rat anti-SOX2	eBioscience/Invitrogen	14-9811-82	1:300
Rabbit anti-FOXA2	Abcam	ab108422	1:300
Rabbit anti-GATA6	Cell Signaling Technology	5851	1:300
Rabbit anti-LEF1	Cell Signaling Technology	2230	1:300
Rabbit anti-CDX2	Cell Signaling Technology	3977	1:300
Goat anti-Brachyury (TBXT)	R&D Systems	AF2085	1:300
Goat anti-SOX17	R&D Systems	AF1924	1:300

Secondary antibodies	Source	Cat#	Dilution
Donkey anti-rat Alexa Fluor 488	Invitrogen	A-21208	1:500
Donkey anti-rat Alexa Fluor 647	Invitrogen	A78947	1:500
Donkey anti-rabbit Alexa Fluor Plus 488	Invitrogen	A32790	1:500
Donkey anti-rabbit Alexa Fluor 555	Invitrogen	A-31572	1:500
Donkey anti-goat Alexa Fluor 488	Invitrogen	A-11055	1:500
Donkey anti-goat Alexa Fluor 647	Invitrogen	A-21447	1:500
Hoechst 33342	Thermo Scientific	62249	1:1,000
