# Supplementary material for: Simplified in vitro generation of human gastruloids for modelling early development
Source: Development. 2026 Oct 23;153(20):dev205719. doi: 10.1242/dev.205719 (PMC13723424; doi:10.1242/dev.205719)
Supplement: Supplementary file 2 [file develop-153-205719_review_history.pdf]

Simplified *in vitro* generation of human gastruloids for modelling early development

Takuya Azami, E. Elizabeth Patton and Jennifer Nichols
DOI: 10.1242/dev.205719

Editor: Matthias Lutolf

Review timeline

**Original submission:**	**24 September 2025**
**Editorial decision:**	**18 November 2025**
**Resubmission:**	**7 April 2026**
**Editorial decision:**	**19 May 2026**
**First revision:**	**3 August 2026**
**Editorial decision:**	**19 August 2026**
**Second revision:**	**24 August 2026**
Accepted:	**31 August 2026**

Original submission

First decision letter

MS ID#: dev.205267

MS Title: Simplified In Vitro Generation of Human Gastruloids for Modelling Early Development

Authors: Takuya Azami; E Elizabeth Patton; Jennifer Nichols

Article Type: Techniques and Resources Report

Dear Dr Nichols,

I have now received the referees reports on the above manuscript, and have reached a decision. The referees' comments are appended below.

As you will see from their reports, the referees recognise the potential of your work, but they also raise significant concerns about it. Given the nature of these concerns, I am afraid I have little choice other than to reject the paper at this stage.

However, having evaluated the paper, I do recognise the potential importance of this work. I would therefore be prepared to consider as a new submission an extension of this study that contains new experiments, data and discussions and that address fully the major concerns of the referees. The work required goes beyond a standard revision of the paper. Please bear in mind that the referees (who may be different from the present reviewers) will assess the novelty of your work in the context of all previous publications, including those published between now and the time of resubmission.

Reviewer 1: SUMMARY OF THE ADVANCE MADE IN THIS PAPER AND ITS POTENTIAL SIGNIFICANCE TO THE FIELD

© 2026. Published by The Company of Biologists under the terms of the Creative Commons Attribution License (https://creativecommons.org/licenses/by/4.0/).

In this study, Azami et al. investigate the variability in human gastruloid protocols, with a particular focus on the CHIR pretreatment step. They identify colony density as a major source of heterogeneity and address this by shifting the CHIR treatment window to the first 24 hours of aggregation. This modification leads to reproducible gastruloid formation, which they validate through morphological quantification and marker staining. While this work represents an important step toward improving the human gastruloid protocol, it currently lacks characterization of the gastruloids' cell type composition.

SUGGESTIONS TO AUTHORS

The recipe for home-made N2B27 is missing from the material and methods. Without this, it is difficult to justify their cultures being more affordable compared to E8 media (≈320$ per 500ml).

Do non-capacitated, HNES1 and H9 lines give rise to elongating gastruloids? This is important to show whether this protocol can be generalized to other primed human ESC/iPSC lines.

An important source of irreproducibility in the current human gastruloid model is inter-experimental variability. In Figures 1,3,4, the authors must report the averages of each experiment and plot the standard deviation. They should also perform statistical analysis to validate their observations.

How are the elongated, ovoid, spheroid morphologies assigned? Is it a subjective quantification method or an automated image analysis? There is no information in the materials and methods.

In Figure 2, in CHIR pretreated colonies, FOXA2 is exclusively referred to as an endoderm marker, however, it is first expressed in early mesoderm cells with anterior primitive streak identity. Staining for SOX17 would support their endoderm claims. Alternatively, they can stain for EOMES to confirm anterior primitive streak identity.

In line 30, the authors state "Efficiency of formation of early gastruloids exhibiting the expected regionalization of the three embryonic germ layers was further enhanced by addition of TGFß-inhibitor", however, there is no data supporting this claim. Addition of SB43 seems to be further restricting the diversity, inhibiting cardiac mesoderm formation. There is no evidence for the presence of endoderm in their gastruloids, and thus no confirmation of all three embryonic germ layers. It is essential to demonstrate that their gastruloids contain endodermal cells. The authors could achieve this by staining for markers such as SOX17, FOXA2, or CDH1. Alternatively, single-cell RNA sequencing could provide a more comprehensive assessment of the cellular diversity within their system.

HNES1 and H9 cells are passaged in hypoxia. It is known from mouse gastruloids that hypoxia preculture promotes spontaneous gastruloid elongation (López-Anguita et. al., Development, 2022). Can they speculate on the role of hypoxia in their human gastruloid protocol? In a more recent paper, the same group reports that early exposure to hypoxia enriches gut endoderm composition in gastruloids (Balaskas et. al. Biorxiv, 2025), however authors do not report any endoderm in their gastruloids. Can they speculate on this?

Improving reproducibility does not appear to extend the longevity of the gastruloids, which can be cultured up to 96 hours. Did the authors attempt to maintain the gastruloids for longer periods? Additionally, what are the effects of embedding the gastruloids in Matrigel? Does this promote somite formation?

In Figure 4d, GATA6-positive rosettes are observed at the posterior region of the gastruloid. What is the identity of these cells? Additionally, SB43 treatment appears to increase SOX2 expression, extending to the anterior-most region. Are these cells all of neural origin? Co-staining with SOX1 could help clarify this.

Reviewer 2: SUMMARY OF THE ADVANCE MADE IN THIS PAPER AND ITS POTENTIAL SIGNIFICANCE TO THE FIELD

© 2026. Published by The Company of Biologists under the terms of the Creative Commons Attribution License (https://creativecommons.org/licenses/by/4.0/).

In this manuscript, Azami and colleagues investigate strategies to improve the efficiency and cost-effectiveness of generating human gastruloids. This topic is relevant, as human gastruloid systems remain less efficient and more variable compared to mouse models. The study begins by comparing commercial N-Diff227 medium with an in-house N2B27 formulation, a reasonable approach given previous observations that in-house media can reduce batch variability and improve efficiency in mouse gastruloids. The authors then test two human pluripotent stem cell lines (pHNES1 and pH9), exploring the influence of cell culture density and Activin A concentration. They report that lower Activin A concentrations correlate with more robust gastruloid formation. The manuscript further examines whether gastruloids can elongate without CHIR pre-treatment and investigates the role of TGFß signaling in this process. While the overall goal to develop an optimized, standardized, and cost-effective protocol, is critical to the field, key aspects of the experimental design and analysis limit the strength of the conclusions.

SUGGESTIONS TO AUTHORS
While the manuscript addresses an important question, the current data do not sufficiently support the main conclusions. The experiments are limited in scope, quantitative analyses are lacking, and key claims are drawn from observations performed predominantly on a single cell line. For these reasons, I do not recommend the manuscript for publication in its current form.
1. Use of multiple human pluripotent stem cell lines. The abstract states that two stem cell lines are used as examples. However, most experiments are performed only in pHNES1. The pH9 line appears to be used only in isolated figures, and quantitative evaluation of the final optimized condition (AF3×) is not shown for both lines. To support the claim of a "standardized platform," the optimized protocol should be demonstrated and quantified across multiple independent pluripotent stem cell lines.

2. Insufficient quantitative and statistical analysis. The primary readout is categorical scoring ("Elongated," "Ovoid," "Spherical," "Degenerated"). No variability across batches is shown, and no quantitative morphometric or molecular metrics accompany the classification. Including statistical analyses and depicting batch-to-batch variability would substantially strengthen the conclusions and address reproducibility.

3. Experimental logic regarding Activin and CHIR treatments. The manuscript repeatedly tests the effect of lower Activin concentrations after having already identified AF3× as the optimal condition earlier in the study. In Figure 3, direct comparison of CHIR pre-treatment versus no pre-treatment under the optimized Activin condition is missing, making it difficult to assess the necessity of this step.

4. Immunofluorescence shown without quantitative support. Immunofluorescence images of gastruloids are included at several points, but no quantification of marker expression or variability is provided. Reporting the proportion of gastruloids exhibiting a given pattern, or quantitative spatial analysis, would strengthen the interpretation.

Given these issues, the current manuscript does not yet provide sufficient experimental evidence or quantitative analysis to support its central claims. I therefore recommend rejection at this stage. If the authors address the concerns listed above, including expanding validation to multiple pluripotent lines and adding robust quantitative and statistical analyses, the work may become suitable for reconsideration.

Resubmission

Response to reviewers

Comments from the Reviewers:

Reviewer 1: SUMMARY OF THE ADVANCE MADE IN THIS PAPER AND ITS POTENTIAL SIGNIFICANCE TO THE FIELD
In this study, Azami et al. investigate the variability in human gastruloid protocols, with a particular focus on the CHIR pretreatment step. They identify colony density as a major source of

© 2026. Published by The Company of Biologists under the terms of the Creative Commons Attribution License (https://creativecommons.org/licenses/by/4.0/).

heterogeneity and address this by shifting the CHIR treatment window to the first 24 hours of aggregation. This modification leads to reproducible gastruloid formation, which they validate through morphological quantification and marker staining. While this work represents an important step toward improving the human gastruloid protocol, it currently lacks characterization of the gastruloids' cell type composition.

We thank the reviewer for considering our 'short techniques and resources report' as an important step toward improving the human gastruloid protocol, which was prompted by the generally poor reproducibility experienced by many researchers interested in using human gastruloids to model gastrulation, organogenesis and developmental disorders. We are grateful for the encouragement to include more characterisation of the composition of our gastruloids as well as examples of more advanced gastruloids showing consistency and specific lineage segregation.

SUGGESTIONS TO AUTHORS

The recipe for home-made N2B27 is missing from the material and methods. Without this, it is difficult to justify their cultures being more affordable compared to E8 media (≈320$ per 500ml).

We are sorry that we originally included only the references for the papers in which the recipe for N2B27 was previously published. We are grateful for the reviewer's suggestion to include the full protocol in our methods section. and have therefore added it as Supplementary Table 1 for convenience to the user. Below we present the costs at the time of writing for the various media that have been used by other researchers for human gastruloid production for the reviewer's information.

Medium	Source, Cat#	Cost/500 ml Catalogue price
**N2B27+A3FX**	***Prepared in house***	**£163.9**
NDiff® 227*	TaKaRa, Y40002	£229
NutriStem® XF	Sartorius, 5-100-1A	£303.00
mTeSR™ Plus	STEMCELL Technologies, 100-0276	£332
Essential 8™ Medium	Gibco, A1517001	£257.65
StemFlex™ Medium	Gibco, A3349401	£317.65

Do non-capacitated, HNES1 and H9 lines give rise to elongating gastruloids? This is important to show whether this protocol can be generalized to other primed human ESC/iPSC lines.

We apologise for our failure to explain capacitation in our report. As described by Rostovskaya and co-workers (see https://w.ncbi.nlm.nih.gov/pubmed/30944104), capacitation is the term chosen to describe the transition required to drive cells from the naïve state of pluripotency into the primed state, which is the starting condition for human gastruloid formation. This can be achieved efficiently by culturing naïve pluripotent hPSCs in N2B27 medium, supplemented with either the tankyrase inhibitor, XAV, or the porcupine inhibitors IWP2 or C59. We have rewritten our manuscript to clarify this confusion and included the following sentence in the methods section: 'Naïve HNES1 were capacitated to the primed state according to the previously reported protocol in which N2B27 basic medium is supplemented with 2 mM of the tankyrase inhibitor, XAV939 (Rostovskaya et al., 2019)'.

An important source of irreproducibility in the current human gastruloid model is inter-experimental variability. In Figures 1,3,4, the authors must report the averages of each experiment and plot the standard deviation. They should also perform statistical analysis to

© 2026. Published by The Company of Biologists under the terms of the Creative Commons Attribution License (https://creativecommons.org/licenses/by/4.0/).

validate their observations.

We thank the reviewer for pointing out this omission and have added the required information and statistics to the relevant experiments as supplementary material.

How are the elongated, ovoid, spheroid morphologies assigned? Is it a subjective quantification method or an automated image analysis? There is no information in the materials and methods.

We apologise for this oversight. In response to the reviewer's excellent suggestion, we have implemented a system to assess more accurately the dimensions and condition of each gastruloid. Examples of gastruloids for each morphology are now presented in Supplementary Figure 1A and the details of the morphological quantification process are included in the materials and methods section, page 11.

In Figure 2, in CHIR pretreated colonies, FOXA2 is exclusively referred to as an endoderm marker, however, it is first expressed in early mesoderm cells with anterior primitive streak identity. Staining for SOX17 would support their endoderm claims. Alternatively, they can stain for EOMES to confirm anterior primitive streak identity.

We apologise for this lack of clarity, so have performed SOX17 staining on a sample of gastruloids (new Figure 4E), which we hope confirms our claim that our gastruloids are able to produce endoderm.

In line 30, the authors state "Efficiency of formation of early gastruloids exhibiting the expected regionalization of the three embryonic germ layers was further enhanced by addition of TGFß-inhibitor", however, there is no data supporting this claim.

We apologise for this oversight and have performed additional experiments and expanded the text to clarify the role of TGFß-inhibitor in standardising lineage segregation in developing gastruloids:

'Although exhibiting apparently similar properties in monolayer culture compared with pHNES1 (Fig. S6A,B), pH9 cells were less efficient and more variable during gastruloid elongation when using the direct aggregation protocol (Fig. S6C-F; Fig. S7). To improve elongation efficiency, we introduced the TGFß inhibitor SB431542 (SB43) after aggregation (Fig. S6C), as SB43 has previously been used to promote gastruloid formation from H9 hPSCs (Hamazaki et al., 2024). SB43 significantly improved the efficiency of elongated gastruloid formation from H9 hPSCs cultured in A3FX; in A20FX, most aggregates became ovoid when SB43 was added (Fig. S6D,E). Interestingly, although the consistent shape and integrity of the H9 gastruloids was improved with SB43, GATA6 and SOX17 expression were absent (Fig. S6F,G), suggesting that endoderm differentiation is not fully specified at 24 hours of differentiation and can be inhibited by SB43 treatment between 24 and 48 hours after aggregation.

To generate elongated gastruloids efficiently, with differentiation into all three germ layers, we optimized the timing of SB43 treatment (Fig. 4A). Administration of SB43 at 1 μM between 48 and 72 hours after aggregation enabled efficient gastruloid elongation for both cell lines (Fig. 4B,C; Fig. S8) while maintaining GATA6+ cardiac mesoderm (Fig. 4D) and SOX17-positive endoderm differentiation (Fig. 4E). Notably, degenerated pH9 gastruloids produced in the absence of SB43 exhibited a broad distribution of GATA6-positive cells throughout the structure, rather than being restricted to the anterior region (Fig. 4D). In contrast, SB43 treatment produced gastruloids with a smoother morphology and anteriorly localized GATA6-positive cells (Fig. 4D). Under these conditions, gastruloids continued to develop beyond 96 hours, and by 144 hours, SOX17- positive cells organized into a tube-like structure resembling a gut tube (Fig. 4E).'

Addition of SB43 seems to be further restricting the diversity, inhibiting cardiac mesoderm formation. There is no evidence for the presence of endoderm in their gastruloids, and thus no confirmation of all three embryonic germ layers. It is essential to demonstrate that their gastruloids contain endodermal cells. The authors could achieve this by staining for markers such as SOX17, FOXA2, or CDH1. Alternatively, single-cell RNA sequencing could provide a more comprehensive assessment of the cellular diversity within their system.

© 2026. Published by The Company of Biologists under the terms of the Creative Commons Attribution License (https://creativecommons.org/licenses/by/4.0/).

We thank the reviewer for this suggestion. We do indeed plan to perform scRNAseq on our human gastruloids from various cell lines, but for that purpose we will extend the culture period to include early organogenesis. This will generate far more data than can be included in a short techniques and resource report, and will probably take at least 6 months, so it will be incorporated in a new manuscript.

HNES1 and H9 cells are passaged in hypoxia. It is known from mouse gastruloids that hypoxia preculture promotes spontaneous gastruloid elongation (López-Anguita et. al., Development, 2022). Can they speculate on the role of hypoxia in their human gastruloid protocol? In a more recent paper, the same group reports that early exposure to hypoxia enriches gut endoderm composition in gastruloids (Balaskas et. al. Biorxiv, 2025), however authors do not report any endoderm in their gastruloids. Can they speculate on this?

We use hypoxia for human gastruloid propagation because it is more akin to the condition experienced in utero, and in our experience, human PSCs grow best under these conditions. We have been encouraged to extend culture of some of our gastruloids to enable clearer identification of specific lineages, particularly endoderm, in our culture conditions. We have clarified the text and added images confirming production of endoderm in our gastruloids.

Improving reproducibility does not appear to extend the longevity of the gastruloids, which can be cultured up to 96 hours. Did the authors attempt to maintain the gastruloids for longer periods? Additionally, what are the effects of embedding the gastruloids in Matrigel? Does this promote somite formation?

We thank the reviewer for pointing out our failure to clarify the longevity of our gastruloids in the text, which we have now corrected. For our initial submission, we focussed on modifying conditions to enhance reproducibility for the elongation step of gastruloid and germ layer specification. In our revised report, we have extended the culture of some gastruloids to illustrate their viability and competence to form specific lineages (see Figure 4 and SF6). The reviewer is quite correct: embedding gastruloids in Matrigel does indeed promote somite formation, which we can achieve by culturing gastruloids beyond 144 h. We are planning a future manuscript in which we analyse later stages of gastruloids and further optimise conditions to achieve somites, gut and neural tube formation in the same aggregate.

In Figure 4d, GATA6-positive rosettes are observed at the posterior region of the gastruloid. What is the identity of these cells? Additionally, SB43 treatment appears to increase SOX2 expression, extending to the anterior-most region. Are these cells all of neural origin? Co-staining with SOX1 could help clarify this.

This is a good question. We are not sure if these structures are rosettes or just aggregations of cells of a particular identity. We thank the reviewer for posing this question, which we plan to address in a future manuscript, including single cell profiling as mentioned above, and more specific immunostaining to identify a range of lineages.

Reviewer 2: SUMMARY OF THE ADVANCE MADE IN THIS PAPER AND ITS POTENTIAL SIGNIFICANCE TO THE FIELD

In this manuscript, Azami and colleagues investigate strategies to improve the efficiency and cost-effectiveness of generating human gastruloids. This topic is relevant, as human gastruloid systems remain less efficient and more variable compared to mouse models. The study begins by comparing commercial N-Diff227 medium with an in-house N2B27 formulation, a reasonable approach given previous observations that in-house media can reduce batch variability and improve efficiency in mouse gastruloids. The authors then test two human pluripotent stem cell lines (pHNES1 and pH9), exploring the influence of cell culture density and Activin A concentration. They report that lower Activin A concentrations correlate with more robust gastruloid formation. The manuscript further examines whether gastruloids can elongate without CHIR pre- treatment and investigates the role of TGFβ signaling in this process. While the overall goal to develop an optimized, standardized, and cost-effective protocol, is critical to the field, key aspects of the experimental design and analysis limit the strength of the conclusions.

© 2026. Published by The Company of Biologists under the terms of the Creative Commons Attribution License (https://creativecommons.org/licenses/by/4.0/).

SUGGESTIONS TO AUTHORS

While the manuscript addresses an important question, the current data do not sufficiently support the main conclusions. The experiments are limited in scope, quantitative analyses are lacking, and key claims are drawn from observations performed predominantly on a single cell line. For these reasons, I do not recommend the manuscript for publication in its current form.

We thank the reviewer for proposing expansion of our short report and have added information accordingly.

1. Use of multiple human pluripotent stem cell lines. The abstract states that two stem cell lines are used as examples. However, most experiments are performed only in pHNES1. The pH9 line appears to be used only in isolated figures, and quantitative evaluation of the final optimized condition (AF3×) is not shown for both lines. To support the claim of a "standardized platform," the optimized protocol should be demonstrated and quantified across multiple independent pluripotent stem cell lines.

For the sake of brevity required for a short report, we focused mainly on the HNES1 cell line, as stated by the reviewer. However, as advised, in our revised manuscript we include more images of gastruloids generated from H9 cells to our supplementary figures.

2. Insufficient quantitative and statistical analysis. The primary readout is categorical scoring ("Elongated," "Ovoid," "Spherical," "Degenerated"). No variability across batches is shown, and no quantitative morphometric or molecular metrics accompany the classification. Including statistical analyses and depicting batch-to-batch variability would substantially strengthen the conclusions and address reproducibility.

As recommended by the reviewer, we have assigned each aggregation an identity, based upon the distinct phenotypes observed, and performed statistical analysis, now added to the methods and supplementary sections to determine the variation between groups and across batches. We are very grateful to the reviewer for this helpful suggestion.

3. Experimental logic regarding Activin and CHIR treatments. The manuscript repeatedly tests the effect of lower Activin concentrations after having already identified AF3× as the optimal condition earlier in the study. In Figure 3, direct comparison of CHIR pre- treatment versus no pre-treatment under the optimized Activin condition is missing, making it difficult to assess the necessity of this step.

We apologise for our inadequate presentation of the assessment of CHIR pre-treatment in optimised Activin conditions. We have added more data and images comparing gastruloids produced without pre-CHIR treatment in reduced Activin conditions to our new Supplementary Figures 6-8.

4. Immunofluorescence shown without quantitative support. Immunofluorescence images of gastruloids are included at several points, but no quantification of marker expression or variability is provided. Reporting the proportion of gastruloids exhibiting a given pattern, or quantitative spatial analysis, would strengthen the interpretation.

We thank the reviewer for this suggestion. However, quantification of marker expression is challenging for gastruloids because of their large solid structure. One option might be to attempt to disaggregate gastruloids and measure the proportions of lineage markers using flow cytometry, but since they are all fixed prior to immunostaining, we fear the protocol would require significant optimisation without much likelihood of success.

Given these issues, the current manuscript does not yet provide sufficient experimental evidence or quantitative analysis to support its central claims. I therefore recommend rejection at this stage. If the authors address the concerns listed above, including expanding validation to multiple pluripotent lines and adding robust quantitative and statistical analyses, the work may become suitable for reconsideration.

© 2026. Published by The Company of Biologists under the terms of the Creative Commons Attribution License (https://creativecommons.org/licenses/by/4.0/).

We thank the reviewer for this excellent suggestion. We agree that making gastruloids from multiple cell lines would be very worthwhile, but would generate more data than can be accommodated in a short technical report, so we are preparing such an article, based upon our recently derived cell lines, separately.

First decision letter

MS ID#: dev.205719

MS TITLE: Simplified In Vitro Generation of Human Gastruloids for Modelling Early Development

AUTHORS: Takuya Azami, E. Elizabeth Patton and Jennifer Nichols

Dear Dr Nichols,

I have now received all the referees reports on the above manuscript, and have reached a decision. The referees' comments are appended below.

The overall evaluation is positive and we would like to publish a revised manuscript in Development, provided that the referees' comments can be satisfactorily addressed. Please attend to all of the reviewers' comments in your revised manuscript and detail them in your point-by-point response. If you do not agree with any of their criticisms or suggestions explain clearly why this is so. If it would be helpful, you are welcome to contact us to discuss your revision in greater detail. Please send us a point-by-point response indicating your plans for addressing the referees' comments, and we will look over this and provide further guidance.

Reviewer 1

SUMMARY OF THE ADVANCE MADE IN THIS PAPER AND ITS POTENTIAL SIGNIFICANCE TO THE FIELD

The authors have successfully addressed the concerns regarding reproducibility and morphological quantification. Furthermore, the addition of SOX17 staining provides convincing evidence of the presence of endoderm within these gastruloids.

SUGGESTIONS TO AUTHORS

Minor comments:

I am afraid the comment "In Figure 2, in CHIR pretreated colonies, FOXA2 is exclusively referred to as an endoderm marker, however, it is first expressed in early mesoderm cells with anterior primitive streak identity. Staining for SOX17 would support their endoderm claims. Alternatively, they can stain for EOMES to confirm anterior primitive streak identity." was misunderstood by the authors. The question was to perform staining in 2D cultures, to answer whether endoderm progrenitors arise "prior" to aggregation in the CHIR pre-treated gastruloids.

Reviewer 2

In this manuscript, Azami and colleagues provide a simplified protocol for the robust generation of human gastruloids using two hESC lines, pHNES1 and pH9. The authors first establish that human gastruloids can be generated, albeit with limited efficiency, using homemade N2B27 medium, which is commonly used for mouse gastruloids. To achieve this, naïve hESCs were capacitated using a CHIR pre-treatment together with low Activin concentrations and a specific cell density. The authors show that these preculture conditions establish a balance of cell lineages that is highly sensitive to both cell density and Activin concentration.

© 2026. Published by The Company of Biologists under the terms of the Creative Commons Attribution License (https://creativecommons.org/licenses/by/4.0/).

The authors then investigated whether this lineage heterogeneity, as well as the CHIR pre-treatment itself, contributed to the limited robustness of gastruloid formation. They show that, without CHIR pre-treatment, gastruloids generated from hESCs cultured in N2B27 with low Activin concentrations could efficiently form gastruloids from pHNES1 cells, although this remained less efficient in pH9 cells. This phenotype was improved by treatment with the TGFß inhibitor SB431542; however, this condition impaired the expression of GATA6 and SOX17, suggesting a loss of endodermal and cardiac mesodermal populations. Delaying SB431542 treatment by 24 hours was sufficient to rescue these lineages, indicating that the timing of TGFß inhibition critically impacts differentiation potential.

This study is timely and overall, well performed. However, a few concerns remain (including some already raised during the initial review process) that should be addressed before publication can be recommended.

SUGGESTIONS TO AUTHORS

Since the initial version, the authors have made a commendable effort to streamline the manuscript, provide additional details regarding experimental outcomes and batch-to-batch variability, and better compare differences between cell lines. I nevertheless have a few comments that I believe would further strengthen the manuscript.

- Figure 4c would benefit from using the same classification scheme for the control condition (without SB431542 treatment).
- Analysis of marker expression patterns in gastruloids: although the authors rightly point out that gastruloids are dense 3D structures that complicate quantitative immunostaining analysis. Documenting the spatial distribution of the markers used across the different experimental conditions would strengthen the conclusions drawn from the representative images shown in the figures. This could potentially be achieved using the "max inscribed circle"-derived axis method already employed in their brightfield imaging analysis. At minimum, the authors should report the number and proportion of gastruloids exhibiting the representative behaviours shown (for example, GATA6 or SOX17 expression patterns).
- In the abstract, the authors continue to describe the method as a "standardized platform." I therefore remain in favour of assessing the success rate of elongated gastruloid formation in additional hESC lines. While I appreciate that this is a short report, inclusion of a supplementary figure showing efficient gastruloid formation in two additional hESC lines under the final optimized conditions would substantially strengthen the claim of a standardized platform without significantly increasing the length of the manuscript. I will, of course, leave it to the editor and authors to determine whether such experiments are feasible.

First revision

Author response to reviewers' comments

Reviewer 1: SUMMARY OF THE ADVANCE MADE IN THIS PAPER AND ITS POTENTIAL SIGNIFICANCE TO THE FIELD
The authors have successfully addressed the concerns regarding reproducibility and morphological quantification. Furthermore, the addition of SOX17 staining provides convincing evidence of the presence of endoderm within these gastruloids.

SUGGESTIONS TO AUTHORS

Minor comments:

I am afraid the comment "In Figure 2, in CHIR pretreated colonies, FOXA2 is exclusively referred to as an endoderm marker, however, it is first expressed in early mesoderm cells with anterior primitive streak identity. Staining for SOX17 would support their endoderm claims. Alternatively, they can stain for EOMES to confirm anterior primitive streak identity." was misunderstood by the

© 2026. Published by The Company of Biologists under the terms of the Creative Commons Attribution License (https://creativecommons.org/licenses/by/4.0/).

authors. The question was to perform staining in 2D cultures, to answer whether endoderm progrenitors arise "prior" to aggregation in the CHIR pre-treated gastruloids.

Thank you for clarifying the point about endoderm differentiation upon CHIR pre-treatment. As suggested, we have immunostained SOX17 in 2D cultured cells after CHIR stimulation, enabling us to demonstrate that SOX17 expression is induced prior to aggregation. We have added a representative image to Fig. S6H.

Reviewer 2: In this manuscript, Azami and colleagues provide a simplified protocol for the robust generation of human gastruloids using two hESC lines, pHNES1 and pH9. The authors first establish that human gastruloids can be generated, albeit with limited efficiency, using homemade N2B27 medium, which is commonly used for mouse gastruloids. To achieve this, naïve hESCs were capacitated using a CHIR pre-treatment together with low Activin concentrations and a specific cell density. The authors show that these preculture conditions establish a balance of cell lineages that is highly sensitive to both cell density and Activin concentration.

The authors then investigated whether this lineage heterogeneity, as well as the CHIR pre-treatment itself, contributed to the limited robustness of gastruloid formation. They show that, without CHIR pre-treatment, gastruloids generated from hESCs cultured in N2B27 with low Activin concentrations could efficiently form gastruloids from pHNES1 cells, although this remained less efficient in pH9 cells. This phenotype was improved by treatment with the TGFβ inhibitor SB431542; however, this condition impaired the expression of GATA6 and SOX17, suggesting a loss of endodermal and cardiac mesodermal populations. Delaying SB431542 treatment by 24 hours was sufficient to rescue these lineages, indicating that the timing of TGFβ inhibition critically impacts differentiation potential.

This study is timely and overall, well performed. However, a few concerns remain (including some already raised during the initial review process) that should be addressed before publication can be recommended.

SUGGESTIONS TO AUTHORS

Since the initial version, the authors have made a commendable effort to streamline the manuscript, provide additional details regarding experimental outcomes and batch-to-batch variability, and better compare differences between cell lines. I nevertheless have a few comments that I believe would further strengthen the manuscript.

- Figure 4c would benefit from using the same classification scheme for the control condition (without SB431542 treatment).

Thank you for this suggestion. We have now included control sample plots using the appropriate classification scheme in Fig. 4C.

- Analysis of marker expression patterns in gastruloids: although the authors rightly point out that gastruloids are dense 3D structures that complicate quantitative immunostaining analysis. Documenting the spatial distribution of the markers used across the different experimental conditions would strengthen the conclusions drawn from the representative images shown in the figures. This could potentially be achieved using the "max inscribed circle"-derived axis method already employed in their brightfield imaging analysis. At minimum, the authors should report the number and proportion of gastruloids exhibiting the representative behaviours shown (for example, GATA6 or SOX17 expression patterns).

We thank the reviewer for this helpful suggestion. We agree that quantitative documentation of marker distribution strengthens the interpretation of the representative immunostaining images, and have therefore performed axial fluorescence intensity profiling of marker expression along the normalized anteroposterior axis of individual gastruloids.

This analysis was applied to the relevant marker combinations and conditions, and the results are now included in Fig. S8C. In this panel, individual gastruloids are shown as thin lines, condition means as thick lines, and shaded regions indicate ± SEM. The number of gastruloids analysed for

© 2026. Published by The Company of Biologists under the terms of the Creative Commons Attribution License (https://creativecommons.org/licenses/by/4.0/).

each condition is also reported. We have also updated the Methods section to describe this quantification approach.

- In the abstract, the authors continue to describe the method as a "standardized platform." I therefore remain in favour of assessing the success rate of elongated gastruloid formation in additional hESC lines. While I appreciate that this is a short report, inclusion of a supplementary figure showing efficient gastruloid formation in two additional hESC lines under the final optimized conditions would substantially strengthen the claim of a standardized platform without significantly increasing the length of the manuscript. I will, of course, leave it to the editor and authors to determine whether such experiments are feasible.

We thank the reviewer for providing this opportunity to extend our repertoire of cell lines to provide a more standardized method for generating gastruloids from various hPSC lines. We applied our protocol to generate gastruloids from Shef6 (Aflatoonian et al., 2010: PMID 20224972), established in the UK as primed hPSC from blastocysts, and pHNES3 (Guo et al., 2016: PMID 26947977), established as naïve hPSC from ICM cells isolated from blastocysts and subsequently capacitated to the primed state. As expected, elongated gastruloids were efficiently generated from Shef6 hPSC with 2 µM SB43 addition between 48h and 72h (80.8%). They exhibited three germ layer differentiation and anterior-posterior axial patterning, confirmed by TBXT, CDX2, SOX2, SOX17, and GATA6 expression. Our attempts to generate elongated gastruloids from pHNES3 cells were unsuccessful, but we decided to include this data since, as published previously, HNES3 (a female line) was found to comprise a mixture of normal cells and those carrying a partial trisomy of chromosome 22 (Guo et al., 2016: PMID 26947977). This is likely to explain the failure in elongation, so could potentially provide a simple means to check developmental potential of a cell line before embarking upon a series of experiments. We include the data in Fig. S9.

Second decision letter

MS ID#: dev.205719R1

MS TITLE: Simplified In Vitro Generation of Human Gastruloids for Modelling Early Development

AUTHORS: Takuya Azami; E. Elizabeth Patton; Jennifer Nichols

Dear Dr Nichols,

I have now received all the referees reports on the above manuscript, and have reached a decision. The referees' comments are appended below.

The overall evaluation is positive and we would like to publish a revised manuscript in Development, provided that the referees' comments can be satisfactorily addressed. Please attend to all of the reviewers' comments in your revised manuscript and detail them in your point-by-point response. If you do not agree with any of their criticisms or suggestions explain clearly why this is so. If it would be helpful, you are welcome to contact us to discuss your revision in greater detail. Please send us a point-by-point response indicating your plans for addressing the referees' comments, and we will look over this and provide further guidance.

Reviewer 2

SUMMARY OF THE ADVANCE MADE IN THIS PAPER AND ITS POTENTIAL SIGNIFICANCE TO THE FIELD

SUGGESTIONS TO AUTHORS

The two additional lines are a valuable addition and clarify the practical scope of the protocol. For Shef6 the authors use 2 µM SB43, whereas pHNES1 and pH9 were treated with 1 µM. The manuscript does not state whether 1 µM was tested on Shef6 and found insufficient; I would ask the authors to

© 2026. Published by The Company of Biologists under the terms of the Creative Commons Attribution License (https://creativecommons.org/licenses/by/4.0/).

report this explicitly, as the negative result at the default concentration is informative for readers applying the protocol to a new line.

On this basis I consider the study genuinely useful for robust and cost-efficient generation of human gastruloids. However, the claim of a standardized platform is not supported by the data, given that inhibitor concentration varies across lines. This does not detract from the study's value: establishing that SB43 concentration is the only parameter requiring adjustment, with cell number, pre-treatment, and media composition holding constant across independently optimized lines, is itself a significant advance for the field than standardization since it tells readers precisely what to vary.

Regarding pHNES5, I would note that an explanation based on chromosome 22 trisomy could not be established from a single line. As this argument is not made in the manuscript itself, I do not consider it an issue for publication.

Once the standardized platform claim is revised, I believe the manuscript will be ready to proceed.

Second revision

Author response to reviewers' comments

Comments from the Reviewers:

Reviewer 2: SUMMARY OF THE ADVANCE MADE IN THIS PAPER AND ITS POTENTIAL SIGNIFICANCE TO THE FIELD

SUGGESTIONS TO AUTHORS
The two additional lines are a valuable addition and clarify the practical scope of the protocol. For Shef6 the authors use 2 μM SB43, whereas pHNES1 and pH9 were treated with 1 μM. The manuscript does not state whether 1 μM was tested on Shef6 and found insufficient; I would ask the authors to report this explicitly, as the negative result at the default concentration is informative for readers applying the protocol to a new line.

We thank the reviewer for pointing out our omission of this important information and giving us the opportunity to add it to the protocol. We have included the following explanation:

'To derive gastruloids from Shef6 PSCs we initially used 1 μM SB43, which is optimal for pHNES1 and pH9. Although elongated Shef6 gastruloids could be generated, the efficiency was lower than for HNES1 or H9 gastruloid production. By increasing the concentration of SB43 to 2 μM, with no other changes to the protocol, Shef6 gastruloids could be produced efficiently.'

On this basis I consider the study genuinely useful for robust and cost-efficient generation of human gastruloids. However, the claim of a standardized platform is not supported by the data, given that inhibitor concentration varies across lines. This does not detract from the study's value: establishing that SB43 concentration is the only parameter requiring adjustment, with cell number, pre-treatment, and media composition holding constant across independently optimized lines, is itself a significant advance for the field than standardization since it tells readers precisely what to vary.

In light of this previously unsuspected variation between cell lines for the concentration of SB43 required for efficient gastruloid elongation, we have moderated our claim that the protocol is 'standardised' and have replaced the word with 'adaptable' the abstract.

Regarding pHNES5, I would note that an explanation based on chromosome 22 trisomy could not be established from a single line. As this argument is not made in the manuscript itself, I do not consider it an issue for publication.

© 2026. Published by The Company of Biologists under the terms of the Creative Commons Attribution License (https://creativecommons.org/licenses/by/4.0/).

We agree with the reviewer that we do not have proper evidence that the failure of pHNES3 to form gastruloids can be attributed to the trisomy, but the suggestion may be of interest to other researchers for whom some cell lines do not readily form gastruloids.

Once the standardized platform claim is revised, I believe the manuscript will be ready to proceed.

Third decision letter

MS ID#: dev.205719R2

MS TITLE: Simplified In Vitro Generation of Human Gastruloids for Modelling Early Development

AUTHORS: Takuya Azami; E. Elizabeth Patton; Jennifer Nichols

Dear Dr Nichols,

I have now received all the referees reports on the above manuscript, and have reached a decision. The referees' comments are appended below.

The overall evaluation is positive and we would like to publish a revised manuscript in Development, provided that the referees' comments can be satisfactorily addressed. Please attend to all of the reviewers' comments in your revised manuscript and detail them in your point-by-point response. If you do not agree with any of their criticisms or suggestions explain clearly why this is so. If it would be helpful, you are welcome to contact us to discuss your revision in greater detail. Please send us a point-by-point response indicating your plans for addressing the referees' comments, and we will look over this and provide further guidance.

© 2026. Published by The Company of Biologists under the terms of the Creative Commons Attribution License (https://creativecommons.org/licenses/by/4.0/).
